# A spatial and projection-based transcriptomic atlas of paraventricular hypothalamic cell types

**DOI:** 10.21203/rs.3.rs-7895391/v1

**Published:** 2025-10-21

**Authors:** Jon Resch, Yuxi Li, Trevor Butler, Stefano Nardone, Christopher Jacobs, Amelia Douglass, Joseph Madara, Miriam McDonough, Jenkang Tao, Elijah Lowenstein, Luhong Wang, Deepti Pant, Samuel Walker, Annette Wang, Harini Srinivasan, Zongfang Yang, John Campbell, Linus T. Tsai, Bradford Lowell

**Affiliations:** University of Iowa; University of Iowa; University of Iowa; Beth Israel Deaconess Medical Center; Beth Israel Deaconess Medical Center; Beth Israel Deaconess Medical Center; Beth Israel Deaconess Medical Center; University of Iowa; Beth Israel Deaconess Medical Center; Beth Israel Deaconess Medical Center; Beth Israel Deaconess Medical Center; Beth Israel Deaconess Medical Center; Beth Israel Deaconess Medical Center; Beth Israel Deaconess Medical Center; Beth Israel Deaconess Medical Center; Beth Israel Deaconess Medical Center; University of Virginia; BIDMC; Beth Israel Deaconess Medical Center

## Abstract

The paraventricular hypothalamus (PVH) controls many behavioral and physiologic processes, including appetite, social behavior, autonomic outflow, and pituitary hormone secretion. However, molecular markers for centrally-projecting PVH neuron populations remain largely undefined, and a complete census of PVH cell types has not been established. Therefore, we performed extensive single-cell/nucleus RNA sequencing to catalog PVH neuron subtypes and multiplexed error-robust fluorescence in situ hybridization (MERFISH) to map them spatially. Our spatial transcriptomic atlas resolves 26 Sim1+ and 29 GABAergic neuron populations from the PVH and surrounding areas, revealing multiple subtypes not described previously and distinct transcriptional programs between neuroendocrine and centrally-projecting neurons. Additionally, projection-based profiling determined neuronal subtypes that project to the parabrachial region (PB) and spinal cord, helping to identify PVH populations that regulate satiety and sympathetic nervous system activity, respectively. Notably, activation of PB-projecting PVH neurons expressing bombesin-like receptor 3 (Brs3) reduces food intake and silencing them causes obesity. Together, this atlas contributes high-resolution PVH spatial and circuit-based gene expression profiles, representing a valuable resource for the field of homeostasis.

## Introduction

The paraventricular hypothalamus (PVH) is among the most functionally diverse and anatomically complex regions of the brain. Essential for maintaining homeostasis, the PVH integrates information about the internal state and external environment, and accordingly adapts endocrine, autonomic, and behavioral outputs^1–3^. PVH neurons are typically classified based on cytoarchitectural subdivisions, projections, and neuroendocrine hormone expression^1–9^. PVH parvicellular neuron projections to the median eminence release hormones into the hypophyseal portal system that then cause release of anterior pituitary hormones to regulate the stress response, thyroid function, and growth, whereas PVH magnocellular neuron projections to the posterior pituitary release vasopressin and oxytocin directly into the systemic circulation^1,3^. Centrally-projecting PVH neurons, on the other hand, are a highly heterogeneous and poorly defined class of PVH neurons innervating regions of the hypothalamus, midbrain, hindbrain, and spinal cord to mediate autonomic and behavioral responses^10^. Despite their importance, the molecular and functional diversity of centrally-projecting PVH neurons remains unresolved.

Among their many functions, the centrally-projecting PVH neurons are well-known for regulating energy balance. PVH neurons that express the melanocortin 4 receptor (*Mc4r*) are crucial for body weight control as their activation reduces food intake, while loss of function causes hyperphagia and obesity^11–15^. Notably, several other PVH neurons have been reported to decrease food intake^15–21^, including prodynorphin (*Pdyn*)-expressing neurons, which, like PVH^*Mc4r*^ neurons, regulate feeding behavior via projections to the parabrachial region (PB). These two populations are distinct, however, because their simultaneous inhibition causes additive effects on hyperphagia and obesity^12,21^. In contrast, the PVH oppositely regulates feeding behavior via neurons expressing thyrotropin-releasing hormone (*Trh*) and pituitary adenylate cyclase-activating peptide (*Adcyap1*) that induce hunger through activation of agouti-related peptide (AgRP) neurons in the arcuate nucleus (ARC)^22^ – highlighting the complexity of appetite regulation by the PVH. Besides appetite, the PVH also controls energy expenditure through nitric oxide synthase 1 (*Nos1*)*-* and brain-derived neurotrophic factor (*Bdnf*)-expressing neuron projections to the spinal cord that drive sympathetic nervous system output to adipose tissue^19,23,24^. That said, because these previously described genetic markers are expressed across multiple PVH neuron subpopulations, the exact transcriptional identity of energy balance-regulating neurons remains unclear, and the lack of precise markers limits our ability to study their regulation and function selectively.

Recent studies characterizing PVH neurons at the molecular level represent an important step towards understanding the diversity of cell types present^25–27^. However, the power of these studies has been limited by sample size and the inability to resolve their spatial organization. Moreover, large-scale single-cell and spatial transcriptomic studies of the entire mouse brain^28–31^ or hypothalamus^32^ lack detailed analysis of PVH neuron subtypes, leaving significant gaps in our understanding of the molecular heterogeneity of PVH neurons. To address these limitations, we employed single-cell/nucleus RNA sequencing (sc/snRNA-seq) and multiplexed error-robust fluorescence *in situ* hybridization (MERFISH) to generate a comprehensive spatial transcriptomic atlas of the PVH at single-cell resolution. This approach uncovered novel marker genes for neuroendocrine populations and revealed molecular signatures of previously unidentified centrally-projecting PVH neurons. Further, we sequenced spinal cord- and PB-projecting PVH neurons to identify marker genes for neurons controlling sympathetic nervous system activity and feeding behavior, respectively. Leveraging this information, we show that stimulation of bombesin-like receptor 3 (*Brs3*)-expressing PVH neuron projections to the PB reduces food intake, PVH^*Brs3*^ neurons are downstream of AgRP neurons, and their silencing promotes weight gain. This atlas serves as a foundational resource for understanding the molecular architecture of the PVH and lays the groundwork for future investigations into the functional roles of its diverse neuronal populations.

## Results

### Molecular profiling of the paraventricular hypothalamus.

**Figure 1:**
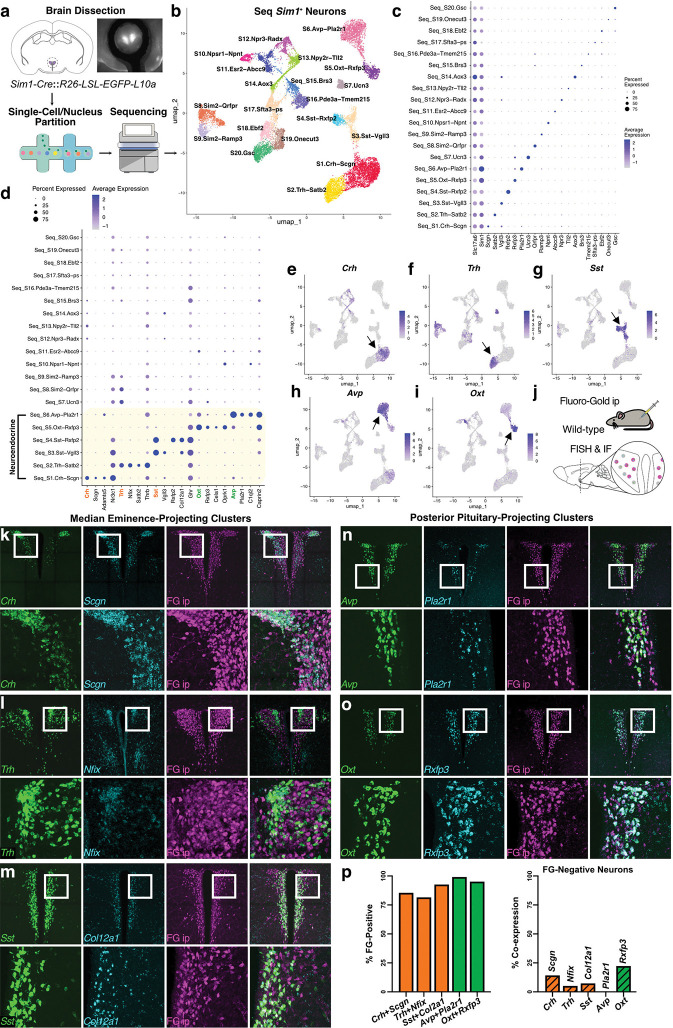
Single-cell/nucleus transcriptional profiling of *Sim1*^+^ neurons. **a**) Illustration of sc/snRNA-seq workflow. **b**) *Sim1*^+^ UMAP comprised of 16,598 cells/nuclei integrated from this study and previously published data from the Allen Brain Cell Atlas. **c**) Dot plot showing the expression of *Slc17a6*, *Sim1*, and marker genes for *Sim1*^+^ clusters calculated using the *Wilcoxon Rank Sum test followed by Bonferroni correction for multiple comparisons*. **d**) Dot plot showing the expression of top marker genes for neuroendocrine clusters. **e-i**) Feature plots depicting the expression of *Crh* (**e**), *Trh* (**f**), *Sst* (**g**), *Avp* (**h**), and *Oxt* (**i)**. **j**) Experimental schematic for retrograde labeling of neuroendocrine neurons with intraperitoneal (ip) injections of Fluoro-Gold (FG) together with fluorescent *in situ* hybridization (FISH) for top neuroendocrine marker genes. **k-o**) Immunofluorescence (IF) for ip-injected FG and FISH of marker genes of neuroendocrine clusters. Median eminence-projecting (left) - *Crh* and *Scgn* (**k**), *Trh* and *Nfix* (**l**), *Sst* and *Col12a1* (**m**). Posterior pituitary-projecting (right) - *Avp* and *Pla2r1* (**n**), *Oxt* and *Rxfp3* (**o**). The lower panels represent magnified views of the boxed regions highlighted in the upper panels. p) Percentage of neurons that co-express distinct neuroendocrine gene pairs that are also labeled by ip-injected FG (left) and percentage of FG-negative neurons that co-express neuroendocrine marker gene pairs (right).

To classify PVH cell types based on their genome-wide expression patterns, we performed single-cell RNA-seq using Drop-seq^33^, and single-nucleus RNA-seq using DroNc-seq^34^ and the 10X Chromium platform on adult male and female mice (**Extended Data Fig. 1a,b**). For each approach, we micro-dissected the PVH region from *Sim1*-Cre^14^::L10-GFP^22^ or wild-type mice (Fig. 1a). After droplet generation, library preparation, sequencing, data pre-processing, and quality control, downstream sc/snRNA-seq analyses were performed using Seurat version 5^35,36^, integrating by sequencing run (“batch”), to generate an atlas of 42,948 cells/nuclei from the PVH and immediately surrounding regions. Cell type clusters were visualized with uniform manifold approximation and projection (UMAP) and annotated using canonical cell type marker genes previously reported in the literature, revealing nearly 80% neurons, with the remaining cells forming distinct populations of non-neuronal/glial cells (**Extended Data Fig. 1c-e; Supplementary Table 1**). We next examined the effects of sc/snRNA-seq technology and sex on cell clustering. While gene and cell type detection differed somewhat between the droplet-based sc/snRNA-seq methods, cells/nuclei from both sexes and all technologies were represented in all clusters (**Extended Data Fig. 1f-i**).

To gain specific insight into PVH neuron diversity, we next reclustered 33,644 neuronal cells/nuclei, which produced a UMAP with clusters predominantly segregated into inhibitory neurons expressing the vesicular GABA transporter (*Slc32a1*; VGAT) and excitatory neurons expressing the vesicular glutamate transporter 2 (*Slc17a6*; VGLUT2) (**Extended Data Fig. 2a-d,g,h; Supplementary Table 2**). We also observed further segregation of excitatory neurons into those expressing the PVH marker gene *Sim1* or the thalamic marker gene *Tcf7l2* (**Extended Data Fig. 2b,e,f,I,j**). Histological assessment confirmed that *Slc32a1* is expressed primarily in areas surrounding the PVH^37,38^, *Sim1* is predominantly expressed within the PVH^39^, and *Tcf7l2* expression is constrained to thalamus dorsal to the PVH^31,40^. We subsequently reclustered glutamatergic and GABAergic neurons separately, resulting in 22 excitatory clusters from 18,920 glutamatergic cells/nuclei (**Extended Data Fig. 2k,l; Supplementary Table 3**) and 28 inhibitory populations from 13,075 GABAergic cells/nuclei surrounding the PVH (**Extended Data Fig. 2m,n; Supplementary Table 4**). Finally, to specifically investigate PVH neuron gene expression profiles, we reclustered only neurons from *Sim1*-positive populations. At this point, we also sought to take advantage of publicly available data. To do so, we examined PVH-assigned cells from the “HypoMap” study, an integrated reference atlas of the entire mouse hypothalamus (**Extended Data Fig. 3a-f; Supplementary Table 5**)^32^. However, after integrating 5,119 putative PVH neurons expressing *Sim1* from HypoMap with our study, we observed discrepancies between the data sets (**Extended Data Fig. 3g-i; Supplementary Table 6**). Notably, seven *Sim1*^+^ clusters were comprised almost entirely of neurons from this study (**Extended Data Fig. 3j**), and a large proportion of HypoMap neurons express markers for “peri-PVH” neurons, including *Cabp7*, *Onecut3*^41^, and *Gsc*^42^ (**Extended Data Fig. 3k**). These results suggest that there is inadequate representation of PVH neuron subtypes within the HypoMap study^32^. Thus, we instead integrated *Sim1*^+^ PVH neurons from the Allen Brain Cell (ABC) Atlas^29^, resulting in 9,301*Sim1*^+^ neurons from this study and 7,297 from the ABC Atlas. Analysis after integration identified 20 distinct clusters, each consisting of cells from both studies that we annotated based on the expression of one or more marker genes (Fig. 1b,c; **Extended Data Fig. 3l-n; Supplementary Table 7**). This final sc/snRNA-seq atlas, comprising 16,598 *Sim1*^+^ neurons, greatly surpasses the number of cells previously available from single-cell transcriptomic studies of the PVH, and also provides detailed molecular markers for PVH neuron populations.

### Unique transcriptional profiles of PVH neuroendocrine populations revealed by sc/snRNA-seq

The PVH is home to parvicellular and magnocellular neuroendocrine neurons that are defined by the synthesis and release of one of five well-known hormones, which include corticotropin-releasing hormone (*Crh*), thyrotropin-releasing hormone (*Trh*), somatostatin (*Sst*), arginine vasopressin (*Avp*), and oxytocin (*Oxt*)^1^. In this study, we identified distinct *Sim1*^+^ neuronal clusters that are enriched for these genes annotated as Seq_S1.Crh-Scgn, Seq_S2.Trh-Satb2, Seq_S3.Sst-Vgll3, Seq_S4.Sst-Rxfp2, Seq_S5.Oxt-Rxfp3, and Seq_S6.Avp-Pla2r1 (Fig.1d–i), and hypothesized that these clusters represent the PVH neuroendocrine populations. However, since these pituitary-regulating hormone genes are expressed across multiple PVH neuron clusters, albeit at lower levels, we sought to confirm our neuroendocrine cluster classifications. To label median eminence- and posterior pituitary-projecting PVH neurons, C57BL/6J mice received intraperitoneal (ip) injections of the retrograde tracer Fluoro-Gold, which labels neurons that project outside the blood-brain barrier when administered systemically (Fig. 1j)^1,43,44^. We then performed co-labeling studies for each putative neuroendocrine cluster using fluorescence *in situ* hybridization (FISH) to demonstrate co-expression of neuroendocrine hormones with novel marker genes determined by sc/snRNA-seq, followed by immunofluorescence for Fluoro-Gold. Of note, sc/snRNAseq identified two putative PVH neuroendocrine populations that express *Sst*, Seq_S3.Sst-Vgll3 and Seq_S4.Sst-Rxfp2, the significance of which is unknown as each expresses the growth hormone receptor (*Ghr*), likely to facilitate negative feedback^45^. To assess the neuroendocrine identity of these PVH^*Sst*^ neuron clusters, we performed FISH for *Col12a1,* taking advantage of its enrichment in both clusters (Fig. 1d). Other gene pairs tested were *Crh-Scgn*, *Trh-Nfix*, *Oxt-Rxfp3*, and *Avp-Pla2r1*. In all cases, greater than 80% of neurons co-expressing a neuroendocrine peptide and its corresponding marker gene were also positive for Fluoro-Gold (Fig. 1k–p). This is consistent with prior reports of *Crh* and *Scgn* co-expression in neuroendocrine neurons controlling the hypothalamic-pituitary-adrenal axis^25,27^. Furthermore, Fluoro-Gold negative neurons expressing *Crh*, *Trh*, *Sst*, *Avp*, and *Oxt* rarely co-expressed the corresponding neuroendocrine marker gene determined by sc/snRNA-seq (Fig. 1p). These findings confirm our neuroendocrine classifications and demonstrate that the intersection of neuroendocrine marker gene pairs identified by sc/snRNA-seq enables approaches for gaining selective genetic access to pituitary-regulating PVH neuron populations.

Given that neuroendocrine neurons share a common projection target and release large amounts of neuropeptide hormones into the circulation, we next assessed whether we could identify a shared transcriptional program that differentiates them from centrally-projecting PVH populations. Marker gene analysis revealed a sharp division in transcriptional profiles (**Extended Data Fig. 4a,b; Supplmentary Tables 8,9)**, identifying genes that distinguish neuroendocrine populations (e.g., *Creb3l2*;**Extended Data Fig. 4c,e**) and centrally-projecting neurons (e.g., *Ntng1*; **Extended Data Fig. 4d,e)**. To further characterize these transcriptional differences, we performed Gene Ontology (GO) enrichment analysis on genes upregulated in PVH neuroendocrine and centrally-projecting populations (**Extended Data Fig. 4f,g; Supplementary Tables 10,11**). We found neuroendocrine neurons are most significantly enriched for genes related to ribosomal function and translation, which may be crucial for the synthesis of large quantities of neuropeptides. In contrast, centrally-projecting neurons were strongly enriched for genes related to the formation and regulation of synapses. These findings suggest differences in the signaling machinery of neuroendocrine versus non-neuroendocrine neuron populations. Additional marker gene analysis comparing median eminence-projecting and posterior pituitary-projecting neuroendocrine subtypes also demonstrated transcriptional differences, highlighting *Agtr1a* as a marker for median eminence-projecting (parvicelluar) neurons and *Plekhg1* as a marker for posterior pituitary-projecting (magnocellular) neurons (**Extended Data Fig. 4h-l; Supplementary Tables 12,13**). GO enrichment analysis revealed that the top pathways for median eminence-projecting populations are related to ion channel activity (**Extended Data Fig. 4m; Supplementary Table 14**). Meanwhile, posterior pituitary-projecting populations again showed enrichment for ribosomal function and translation-related pathways, which likely are critical for supporting direct secretion of large quantities of AVP and OXT into the systemic circulation to regulate distant target organs (**Extended Data Fig. 4n; Supplementary Table 15)**^1^.

### Spatial transcriptomic profiling of the PVH with MERFISH.

**Figure 2:**
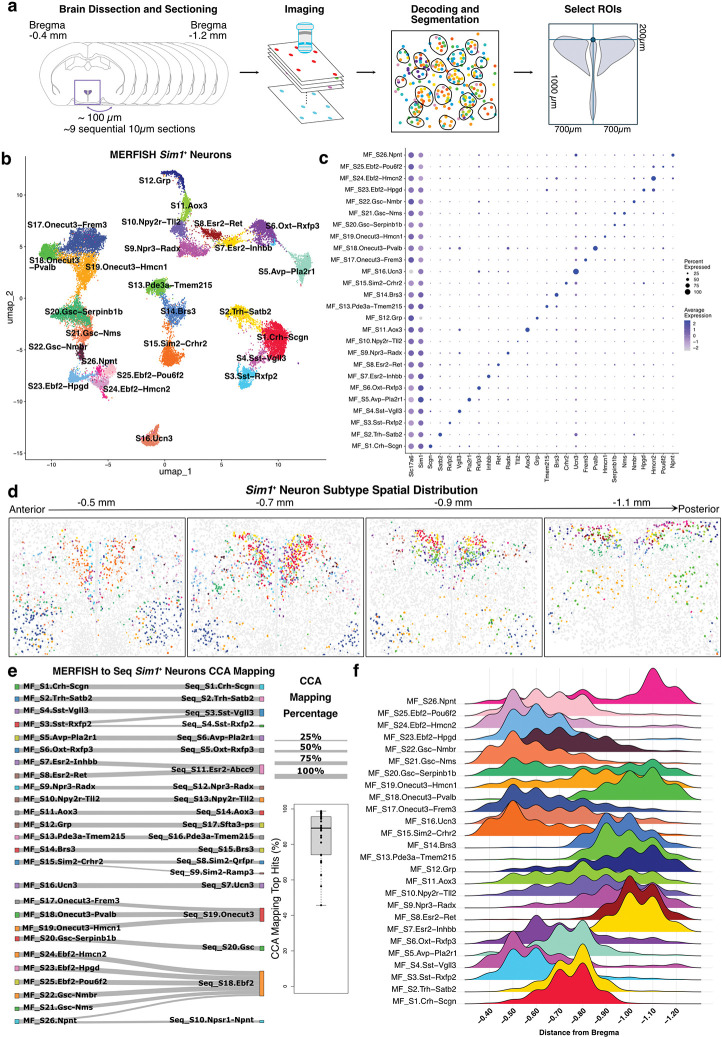
MERFISH spatial transcriptomic profiles of *Sim1*^+^ neurons in the PVH. **a**) Schematic showing key steps in the MERFISH experimental workflow. **b**) UMAP showing 24, 132 *Sim1*^**+**^ neurons. **c**) Dot plot showing the expression of *Slc17a6*, *Sim1*, and marker genes for *Sim1*^**+**^ MERFISH clusters calculated using the *Wilcoxon Rank Sum test followed by Bonferroni correction for multiple comparisons*. **d**) Representative coronal MERFISH sections show the spatial distribution of *Sim1*^**+**^ neuron types from a male mouse (Slide 3), color-coded by cluster identity. **e**) Sankey plot depicting cluster correspondence between MERFISH *Sim1*^**+**^ neuron clusters and sc/snRNA-seq *Sim1*^**+**^ neuron clusters in the PVH (25% cutoff). Line thickness represents the proportion of cells from each MERFISH cluster that is predicted to map to a particular sc/snRNA-seq cluster. Box and whisker plot (right) depicts the upper and lower quartiles of the Canonical Correlation Analysis (CCA) mapping percentages for the top hit from each MERFISH cluster. The black line within the box represents the median CCA mapping percentage, and the whiskers depict 1.5 times the interquartile range. **f**) Ridge plot showing the distribution of *Sim1*^**+**^ neuron clusters along the rostral-to-caudal axis.

Droplet-based sc/snRNA-seq technologies are powerful tools for identifying and characterizing cell type diversity. However, they require tissue dissociation, preventing the retention of spatial information, and may fail to detect functionally important genes expressed at low levels. Therefore, we used MERSCOPE^29,46^, an imaging-based MERFISH platform capable of detecting low-abundance transcripts with single-molecule sensitivity^46–48^, to resolve the spatial organization of the PVH and surrounding regions. We assayed the spatial distribution of 503 genes specifically curated for the PVH region, comprised of top differentially expressed genes identified in our sc/snRNA-seq analyses, canonical marker genes for neuronal and glial populations, and functionally relevant genes selected from the literature (**Supplementary Tables 16,17**). In total, we imaged 41 coronal sections across six mice. Brain sections were collected at intervals of approximately 100 μm along the rostral-caudal axis of the PVH, ranging from approximately 0.4 mm to 1.2 mm caudal to bregma according to the Franklin-Paxinos atlas^49^. After imaging, individual cells were segmented using Cellpose 2.0^50^ and filtered to remove cells with low transcript counts (**Extended Data Fig. 5a**). Then, for each coronal slice, we systematically defined the region of interest (ROI) covering the PVH and peri-PVH and subset the data to retain only cells within these regions (Fig. 2a; **Supplementary Table 18**). After subsetting for the ROI, we were able to perform cell type clustering on 155,546 spatially resolved cells. Our initial all-cell MERFISH clustering comprised eight major cell types, approximately 65% of which were classified as neurons (**Extended Data Fig. 5b-d; Supplementary Table 19**). Importantly, each MERFISH slide contributed proportionally to all major cell type clusters, with no sex-dependent batch effects on clustering observed after data integration, demonstrating the technical replicability of the MERFISH assay across multiple trials (**Extended Data Fig. 5e,f**). Importantly, plotting our MERFISH spatial data using polygons color-coded by major cell type, with neurons divided into excitatory and inhibitory populations, recapitulates the known cellular organization in this region of the hypothalamus (**Extended Data Fig. 5g,h**). Specifically, polygons of excitatory neurons are organized in the distinct triangular distribution of the PVH, while inhibitory neurons surround the PVH. Non-neuronal cell types do not show a particular spatial organization, except for a distinct layer of polygons classified as ependymal cells that line the third ventricle and enrichment of oligodendrocytes in the fornix.

Following initial all-cell MERFISH analysis, we performed subclustering of excitatory (*Slc17a6*^+^) and inhibitory (*Slc32a1*^+^) neurons as we did for sc/snRNA-seq data. Excitatory neurons were further divided based on *Sim1* expression, and the three major neuron types, *Slc17a6*^+^/*Sim1*^+^, *Slc17a6*^+^/*Sim1*^−^ (**Extended Data Fig. 5i,j; Supplementary Table 20**), and *Slc32a1*^+^, were reclustered. To characterize the anatomical location of MERFISH cell types, we next performed spatial domain analysis on all neuron subpopulations using the SpaDo package in R^51^. This computational method integrates gene expression and spatial proximity information from multiple slices, allowing for unbiased anatomical categorization of neurons, which can be used to link the molecular profiles from MERFISH cell types to previously described neuroanatomical PVH subdivisions^1^. Twenty-nine domains were identified distributed across “Rostral” (R1-R11; −0.4 to −0.6 mm from bregma), “Intermediate” (M1-M9; −0.7 to −0.9 mm from bregma), and “Caudal” (C1-C9; −1.0 to −1.2 mm from bregma) regions (**Extended Data Fig. 6a,b; Supplementary Table 21**). Finally, the majority of spatial domains show neuron subtype enrichment, with domains R4, R5, M1, M2, M9, C4, and C7 primarily encompassing *Slc17a6*^+^/*Sim1*^+^ neurons (**Extended Data Fig. 6c; Supplementary Table 22**).

### Spatial distribution of *Sim1*^+^ MERFISH clusters.

MERFISH cell clustering of 24,132 *Sim1*-expressing neurons resulted in the identification of 26 glutamatergic (*Slc17a6*^+^) clusters that we annotated according to the expression of one or more marker genes (Fig. 2b,c; **Supplementary Table 23**). Importantly, plotting *Sim1* expression and *Sim1*^+^ MERFISH clusters confirms the expected spatial enrichment within the PVH (Fig.2d; **Extended Data Fig. 6d**)^31,39^. Next, we performed canonical correlation analysis (CCA) to examine the transcriptional similarity between MERFISH-defined and sc/snRNA-seq-defined *Sim1*^+^ clusters^36^. CCA identified strong correspondence between cells belonging to MERFISH *Sim1*^+^ clusters and those from *Sim1*^+^ sc/snRNA-seq (Fig. 2e; **Supplementary Table 24**). There are, however, a few instances where multiple MERFISH *Sim1*^+^ clusters map to a single sc/snRNA-seq cluster. For example, all MERFISH clusters enriched for *Onecut3*, including MF_S17.Onecut3-Frem3, MF_S18.Onecut3-Pvalb, and MF_S19.Onecut3-Hmcn1 (**Extended Data Fig. 6e**), map to the Seq_S15.Onecut3 cluster. We hypothesize that this is due to the improved gene detection with MERFISH, which increased our resolution of neurons enriched for *Onecut3* expression and produced multiple clusters upon analysis. Overall, there is a general correspondence between *Sim1*^+^ MERFISH and sc/snRNA-seq clusters, enabling the inference of genome-wide expression levels for spatially-resolved neuron populations in the PVH region.

**Figure 3:**
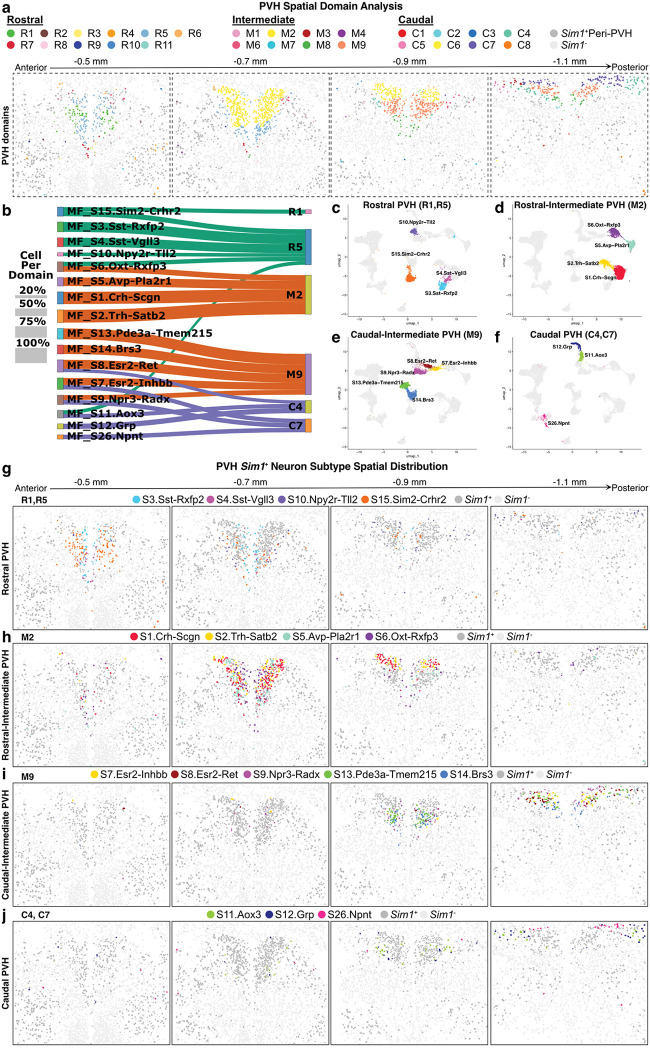
Spatial domain analysis of *Sim1*^+^ MERFISH clusters. **a**) Representative MERFISH sections showing the anatomical distribution of spatial domains enriched for *Sim1*^+^ PVH neurons from a male mouse (Slide 3), color-coded by domain identity. **b**) Sankey plot depicting the proportion of each MERFISH *Sim1*^**+**^ PVH neuron subtype within each spatial domain (20% cutoff). **c-f**) *Sim1*^**+**^ MERFISH reference atlas UMAP highlighted clusters belonging to Rostral (**c**), Rostral-Intermediate (**d**), Caudal-Intermediate (**e**), and Caudal (**f**) PVH neuron spatial groups. **g-j**) Representative coronal sections from a male mouse (Slide 3) illustrating the distribution of *Sim1*^**+**^ MERFISH neuron types in each spatial PVH group.

We next evaluated the spatial location of *Sim1*^+^ clusters from rostral to caudal (Fig. 2f). Using the multi-slice spatial domain analysis performed on all neurons above (**Extended Data Fig. 6a-c**), we delineated PVH and “peri-PVH” *Sim1*^+^ neuron compartments (Fig. 3a–b; **Extended Data Fig. 8a,c**), and PVH neurons were further partitioned into “Rostral,” “Rostal-Intermediate,” Caudal-Intermediate,” and “Caudal” spatial groups (Fig. 3c–j). The Rostral PVH clusters include MF_S3.Sst-Rxfp2, MF_S4.Sst-Vgll3, MF_S10.Npy2r-Tll2, and MF_S15.Sim2-Crhr2, which are primarily located in spatial domains R1 and R5, approximating respectively, the anterior (PVa) and anterior periventricular (PVHpv) parts of the PVH (Fig. 3a–c,g)^1^. As expected, *Sst*^+^ neurons are concentrated in the PVHpv, while MF_S10.Npy2r-Tll2, and MF_S15.Sim2-Crhr2 are located in the PVa. Of interest, single-minded 2 (*Sim2*), a homolog of *Sim1*, marks the MF_S15.Sim2-Crhr2 cluster (**Extended Data Fig. 7a,b,d**). While *Sim1* expression is required for the development of the PVH^23^, disruption of *Sim2* expression causes reductions in the density of *Trh*^*+*^ and *Sst*^*+*^ neurons^52^. *Sim2* is primarily expressed by two distinct *Sim1*^+^ clusters, one of which is the aforementioned MF_S15.Sim2-Crhr2 cluster that also expresses corticotropin-releasing hormone receptor 2 (*Crhr2*) (**Extended Data Fig. 7c-e**). The other is MF_S18.Onecut3-Pvalb, which is located in the caudal ventrolateral Peri-PVH region (**Extended Data Fig. 7d; Extended Data Fig. 8b**). Notably, PVH^*Sim2*^ neurons are not labeled by systemic Fluoro-Gold injection and the MF_S15.Sim2-Crhr2 cluster expresses both *Trh* and *Adcyap1* (**Extended Data Fig. 7d,f**), suggesting that they are the previously described excitatory afferents to ARC^*Agrp*^ neurons that drive feeding^22,53^. MF_S15.Sim2-Crhr2 neurons are also enriched for known drivers of synaptic plasticity, including *Bdnf* ^54^ and cerebellin-2 (*Cbln2*; **Extended Data Fig. 7d**)^55,56^, which is consistent with increased excitatory synapses formed between the PVH and ARC^*Agrp*^ neurons after fasting^53,57^. Indeed, our recent study has revealed that PVH^*Sim2*^ neurons play an important role in hunger regulation^58^. On the other hand, the MF_S10.Npy2r-Tll2 cluster is marked by neuropeptide Y (NPY) Y2 receptor (*Npy2r*) and tolloid-like protein 2 (*Tll2*) (Fig. 2c and Fig. 3c,g), but does not express other NPY receptors. Given thatthe orexigenic effects of NPY in the PVH^59^ are mediated by NPY1R and NPY5R^60^, we speculate MF_S10.Npy2r-Tll2 neurons may be modulated by caloric deficit, but do not regulate food intake.

The Rostral-Intermediate group consists of four MERFISH clusters that correspond to neuroendocrine populations (Fig. 2e), MF_S1.Crh-Scgn, MF_S2.Trh-Satb2, MF_S5.Avp-Pla2r1, and MF_S6.Oxt-Rxfp3 (Fig. 3d,h). All clusters are primarily located in spatial domain M2, but MF_S6.Oxt-Rxfp3 also has a substantial number of neurons located in spatial domain R5, corresponding to the anterior magnocellular part of the PVH (PVHam) (Fig. 3a,b)^1^. Of note, spatial domain analysis did not differentiate parvicelluar and magnocelluar neuroendocrine subtypes previously defined in rats^2,7^. This may be because spatial domain analysis with SpaDo does not incorporate cytoarchitecture; however, parvicellular and magnocellular cells are also difficult to distinguish with Nissl staining alone in mouse^1^.

The Caudal-Intermediate PVH group is comprised of MF_S7.Esr2-Inhbb, MF_S8.Esr2-Ret, MF_S9.Npr3-Radx, MF_S13.Pde3a-Tmem215, and MF_S14.Brs3 clusters located primarily in spatial domain M9, which closely corresponds to the ventral zone of the medial parvicellular (PVHmpv) part of the PVH (Fig. 3a,b,e,i)^1^. Many clusters in this group are marked by genes for hormone and neuropeptide receptors, such as estrogen receptor 2 (*Esr2*) and natriuretic peptide receptor 3 (*Npr3*), which have been reported to regulate stress responses and blood pressure^61–65^. *Esr2* is enriched in two distinct clusters, MF_S7.Esr2-Inhbb and MF_S8.Esr2-Ret (**Extended Data Fig. 7g-i)**, while *Npr3* is primarily expressed by MF_S9.Npr3-Radx neurons located in the intermediate and caudal PVH, which exhibit minimal co-labeling with systemically injected Fluoro-Gold (**Extended Data Fig. 7j-m**). Notably, MF_S14.Brs3 is marked by specific expression of bombesin-like receptor subtype 3 (*Brs3*), an important gene for body weight regulation and metabolism (Fig. 2C and Fig. 3e,i)^66^. Consistent with this, PVH^*Brs3*^ neurons exhibit increased Fos expression following refeeding^67,68^, and chemogenetic manipulation of their activity bidirectionally regulates food intake^67^, similar to PVH^*Mc4r*^ and PVH^*Pdyn*^ neurons. Thus, based on prior work, PVH^*Brs3*^ neurons are of interest for the future study of satiety regulation.

The Caudal PVH group comprises the MF_S11.Aox3, MF_S12.Grp, and MF_S26.Npnt clusters located in spatial domains C4 and C7, which are comparable to the lateral parvicellular (PVHlp) and forniceal (PVHf) parts of the PVH (Fig. 3a,b,f,j). Of interest, MF_S12.Grp cluster is marked by specific expression of gastrin-releasing peptide (*Grp*; Fig. 2c and **Extended Data Fig. 3f,j**), which is decreased in the PVH following fasting and increased by melanocortin signaling, raising the possibility that these neurons may regulate energy balance^69^.

Finally, there are 10 *Sim1*^*+*^ neuronal clusters in the Peri-PVH group (**Extended Data Fig. 8a-c**). While Peri-PVH clusters express *Sim1*, they are located adjacent to the PVH in separate spatial domains (R2, R4, R9, M1, M6, and C6) and have distinct transcriptional characteristics. With the exception of neurons expressing urocortin 3 (*Ucn3*), neuron subtypes in this region are largely of unknown function, and include MF_S16.Ucn3, MF_S17.Onecut3-Frem3, MF_S18.Onecut3-Pvalb, MF_S19.Onecut3-Hmcn1, MF_S20.Gsc-Serpinb1b, MF_S21.Gsc-Nms, MF_S22.Gsc-Nmbr, MF_S23.Ebf2-Hpgd, MF_S24.Ebf2-Hmcn2, and MF_S25.Ebf2-Pou6f2. Consistent with our spatial characterization of these Peri-PVH groups (**Extended Data Fig. 8a,b)**, previous studies have identified *Onecut3*- and *Gsc*-expressing neurons to be located laterally and ventrally to the PVH^41,42^. Moreover, *Ucn3*-expressing neurons are a relatively small population of peri-PVH neurons that extend laterally from the PVH towards the fornix in spatial domain M6 (**Extended Data Fig. 8a-c)** and are involved in stress and parenting behaviors^70,71^.

### Transcriptional similarity of mouse and human PVH neurons.

Previous characterization of PVH neuron populations in human samples has primarily focused on neuroendocrine subtypes^72–74^. To ascertain whether the PVH neuron populations identified in our transcriptomic study resemble those in the human PVH, we performed a comparative analysis between our mouse sc/snRNA-seq atlas and human brain snRNA-seq data. To achieve this, first, we retrieved all cells from dissections containing the PVH from two publicly available human studies^75,76^ and clustered them using Seurat 5. Next, as we did for mouse sc/snRNA-seq clustering of PVH neurons, we subset the data to only include *SIM1*^+^ clusters and reclustered the remaining 3,432 *SIM1*^+^ nuclei, resulting in 21 distinct *SIM1*^+^ neuronal clusters (**Extended Data Fig. 8d,e; Supplementary Table 25**). To estimate the transcriptomic similarity between human and mouse PVH neurons, we performed CCA comparing *Sim1*/*SIM1*-positive clusters, which also allowed us to provide the analogous mouse MERFISH cluster identifiers. Strikingly, we observed a high degree of transcriptional correlation across species, with notable similarity between humans and mice for neuroendocrine hormone-, *Sim2-,* and *Ucn3*-expressing neuron populations (**Extended Data Fig. 8f; Supplementary Table 26**).

### MERFISH atlas of peri-PVH GABAergic neurons

**Figure 4:**
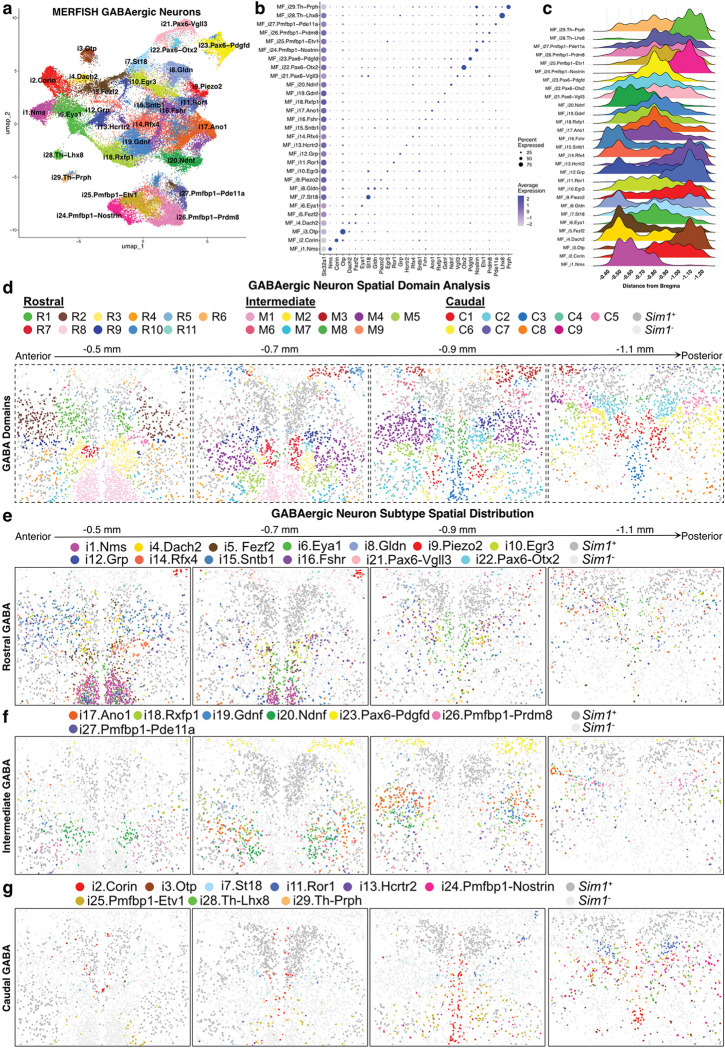
MERFISH spatial transcriptomic profiling of GABAergic neurons surrounding the PVH. **a**) UMAP plot showing 53,294 GABAergic neurons. **b**) Dot plot showing the expression of *Slc32a1* and marker genes for GABAergic neuron clusters calculated using the *Wilcoxon Rank Sum test followed by Bonferroni correction for multiple comparisons*. **c**) Ridge plot showing the distribution of GABAergic neuronal clusters along the rostral-to-caudal axis. **d**) Representative MERFISH sections showing the anatomical distribution of spatial domains enriched for GABAergic neurons from a male mouse (Slide 3), color-coded by domain identity. **e-g**) Representative coronal MERFISH sections showing the Rostral (**e**), Intermediate (**f**), and Caudal (**g**) spatial distribution of 29 GABAergic neuron types from a male mouse (Slide 3), color-coded by cluster identity.

As noted above, the PVH is surrounded by GABAergic (*Slc32a1*^+^) neurons, some of which have been shown to project locally into the PVH^38^ and are proposed to regulate the HPA axis^37,77^. Specific analysis of GABAergic MERFISH populations included 53,294 neurons that clustered into 29 distinct populations. We labeled each cluster according to the expression of one or more marker genes identified through differential gene expression analysis (Fig. 4a,b; **Supplementary Table 27**). Next, we performed CCA between MERFISH and sc/snRNA-seq GABAergic neuron clusters to assess transcriptomic agreement between technologies, and this analysis demonstrated a high degree of similarity (**Extended Data Fig. 9a; Supplementary Table 28**). Finally, we plotted the spatial distribution of the GABAergic MERFISH clusters along the rostral-to-caudal axis, grouping clusters according to spatial domains into “Rostral,” “Intermediate,” or “Caudal” categories (Fig. 4c–g; **Extended Data Fig. 9b**).

Rostral GABAergic neurons include MF_i1.Nms, MF_i4.Dach2, MF_i5. Fezf2, MF_i6.Eya1, MF_i8.Gldn, MF_i9.Piezo2, MF_i10.Egr3, MF_i12.Grp, MF_i14.Rfx4, MF_i15.Sntb1, MF_i16.Fshr, MF_i21.Pax6-Vgll3, and MF_i22.Pax6-Otx2 (Fig. 4d–e). Of these, MF_i1.Nms and MF_i12.Grp represent neurons located in the suprachiasmatic nucleus (SCN; Fig. 4e). Rostral GABAergic neurons also identify subparaventricular zone (SPZ) neuron populations that have been difficult to target previously. Of interest, the SPZ is the major output of the SCN^78^, and SPZ clusters include MF_i5. Fezf2, MF_i6.Eya1, and MF_i14.Rfx4. Intermediate GABAergic clusters include MF_i17.Ano1, MF_i18.Rxfp1, MF_i19.Gdnf, MF_i20.Ndnf, MF_i26.Pmfbp1-Prdm8, and MF_i27.Pmfbp1-Pde11a neurons residing ventral and lateral to the PVH in the anterior hypothalamic area (AHA), and the MF_i23.Pax6-Pdgfd cluster located dorsal to the PVH (Fig. 4f). Finally, the Caudal GABAergic neuron subtypes include MF_i2.Corin and MF_i29.Th-Prph located in the periventricular hypothalamus, the latter of which expresses *Th*, *Ddc*, *Slc18a2*, and *Slc6a3*, suggesting they release dopamine in addition to GABA (Fig. 4g). Remaining Caudal clusters include MF_i3.Otp, MF_i7.St18, MF_i11.Ror1, MF_i13.Hcrtr2, MF_i24.Pmfbp1_Nostrin, MF_i25.Pmfbp1-Etv1, and MF_i28.Th-Lhx8 clusters located in the posterior AHA (Fig. 4g). Together, MERFISH analysis offers the first comprehensive molecular characterization of peri-PVH GABAergic neurons.

### Targeted transcriptomic profiling of spinal cord-projecting PVH neurons.

**Figure 5:**
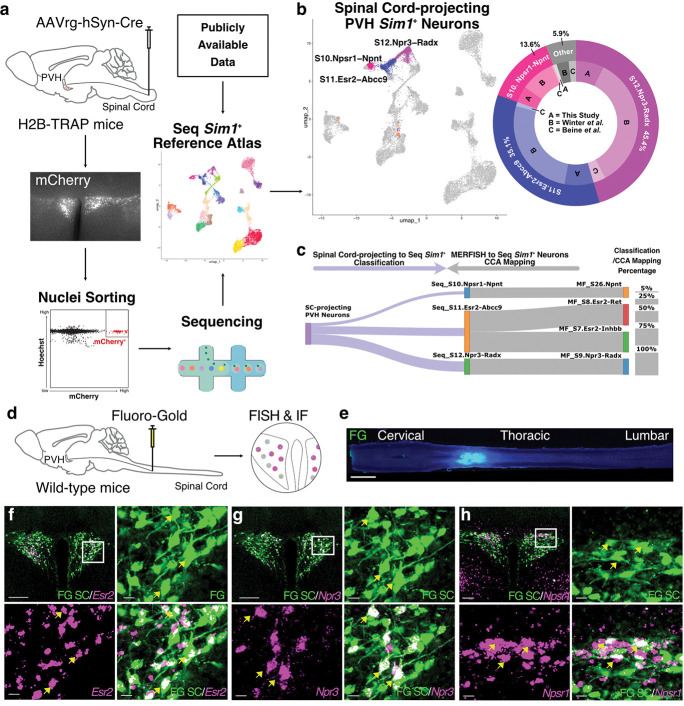
Transcriptomic profiling of spinal cord-projecting PVH *Sim1*^+^ neurons. **a**) Experimental workflow for targeted single-nucleus RNA sequencing of spinal cord-projecting PVH neurons. **b**) Spinal cord-projecting PVH neurons from this study and previously published data^89,90^ were classified via the *Sim1*^**+**^ sc/snRNA-seq reference atlas and projected into its UMAP space. The donut plot illustrates the proportion of spinal cord-projecting PVH neurons that map to individual sc/snRNA-seq *Sim1*^**+**^ neuron clusters annotated by study. **c**) Three-level Sankey plot showing the mapping percentage of spinal cord-projecting PVH neurons classified using the mouse *Sim1*^**+**^ sc/snRNA-seq reference atlas (left mapping to center; 5% cutoff) with corresponding *Sim1*^**+**^ MERFISH clusters identified via canonical correlation analysis (CCA; right mapping to center). The line thickness represents strength of mapping. **d**) Schematic diagram of retrograde Fluoro-Gold (FG) injection into the thoracic spinal cord. Coronal brain sections containing the PVH were collected for Immunofluorescence (IF) and fluorescence *in situ* hybridization (FISH). **e**) Representative longitudinal section of the spinal cord showing the bilateral thoracic injection sites labeled by FG IF. Scale bar = 2 mm. **f-h**) IF for spinal cord-injected FG and FISH for *Esr2* (**f**), *Npr3* (**g**), and *Npsr1* (**h**). For each, the top-left panel shows a low-magnification view of FG and FISH labeling. Remaining panels provide magnified views of the boxed region for FG IF (top-right), FISH (bottom-left), or both (bottom-right). Yellow arrows indicate representative co-labeled neurons. Low magnification scale bar = 100 μm, high magnification scale bar = 20 μm.

Numerous studies have demonstrated that PVH neurons project to the spinal cord^2,4,5,8,9,19,79–84^, many of which are thought to activate sympathetic preganglionic neurons in the intermediolateral cell column to regulate cardiometabolic physiology^19,23,24,80,85–88^. Spinal cord-projecting PVH neurons have been sequenced previously^89,90^; however, prior studies did not profile PVH neurons that project to the thoracic spinal cord, where most sympathetic preganglionic neurons are located, and they did not provide molecular markers that differentiate spinal cord-projecting neurons from other PVH neuron subtypes. Therefore, we profiled PVH neurons that project to the thoracic spinal cord and mapped them onto our *Sim1*^+^ sc/snRNA-seq reference atlas. H2B-TRAP mice^91^ were injected with retrograde AAV-Cre into the thoracic (~T2-T4) spinal cord to selectively label the nuclei of spinal cord-projecting PVH neurons with mCherry for subsequent fluorescence-activated nuclei sorting (FANS; Fig. 5a). After sequencing and clustering, we merged the thoracic spinal cord-projecting *Sim1*^+^ neuron data with *Sim1*^+^ neurons present in previously published spinal cord-projecting datasets^89,90^. Subsequently, we classified the spinal cord-projecting cells based on our *Sim1*^+^ sc/snRNA-seq reference atlas and projected them onto the reference UMAP using the MapQuery function in Seurat 5. Results showed agreement across all studies, suggesting that spinal cord-projecting PVH neurons share transcriptional similarities regardless of the spinal level to which they project, with most clustering within one of three populations: Seq_S10_Npsr1-Npnt (13.6%), Seq_S11_Esr2-Abcc9 (35.1%), or Seq_S12_Npr3-Radx (45.4%) (Fig. 5b, **Supplementary Table 29**). Based on *Sim1*^+^ MERFISH to sc/snRNA-seq CCA mapping, the corresponding MERFISH clusters for spinal cord-projecting populations are MF_S7.Esr2-Inhbb, MF_S8.Esr2-Ret, MF_S9.Npr3-Radx, and MF_S26.Npnt (Fig. 5c).

To confirm the molecular identity of spinal cord-projecting PVH neurons, we injected the retrograde tracer, Fluoro-Gold, into the thoracic spinal cord and subsequently performed FISH for *Esr2*, *Npr3,* or Neuropeptide S receptor 1 (*Npsr1)* (Fig. 5d,e). Our histological analysis revealed colocalization of Fluoro-Gold with the mRNA of all three marker genes we assayed. Notably, the colocalization of *Esr2* and *Npr3* with Fluoro-Gold was predominantly observed in the intermediate and caudal regions of the PVH (Fig. 5f,g), which is consistent with the spatial patterning of these genes identified by MERFISH (**Extended Data Fig. 7i,l**). Likewise, a separate population of Fluoro-Gold-labeled neurons in the caudal PVH was also found to be positive for *Npsr1* mRNA (Fig. 5h), matching the pattern identified by MERFISH (Fig. 3j). Together, these data support that there are three predominant and transcriptionally distinct spinal cord-projecting PVH neuron populations that are likely involved in sympathetic regulation. However, the functional role of each specific spinal cord-projecting PVH population is not known and is an important area of future study.

### Detection of satiety marker genes in *Sim1*^+^ neurons with MERFISH

**Figure 6:**
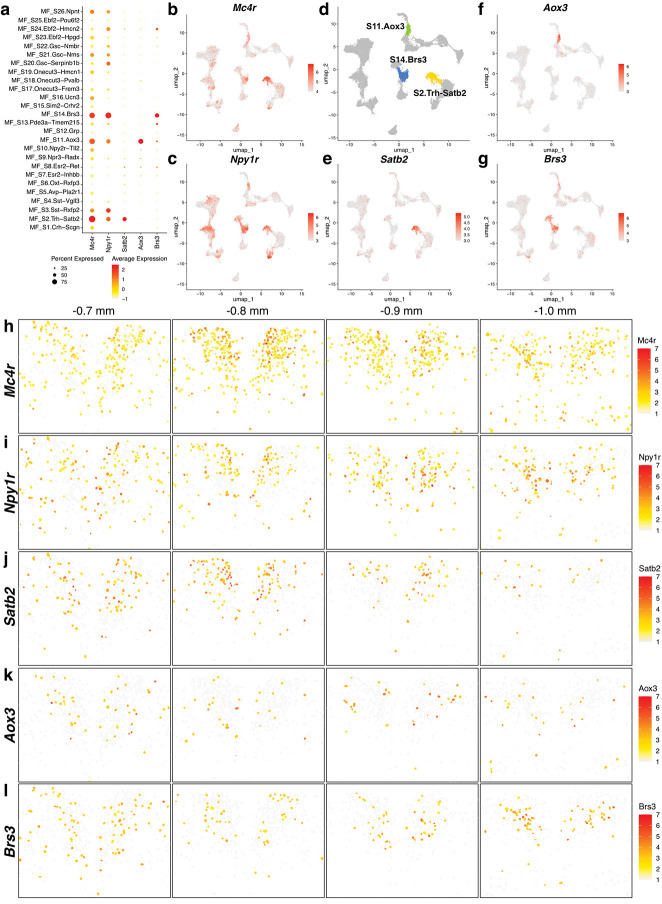
MERFISH characterization of *Mc4r* and *Npy1r* expression in PVH *Sim1*^+^ neurons **a**) Dot plot showing marker genes for *Sim1*^+^ neuron clusters with enriched expression of *Mc4r* and *Npy1r*. **b,c**) Feature plots showing the expression of *Mc4r* (**b**) and *Npy1r* (**c**). **d**) *Sim1*^**+**^ MERFISH reference atlas UMAP highlighting the S2.Trh-Satb2, S14.Brs3, and S11.Aox3 clusters. **E-G**) Feature plots showing the expression of *Satb2* (**e**), *Aox3* (**f**), and *Brs3* (**g**). **h-l**) Image feature plots showing the spatial expression of *Mc4r* (**h**), *Npy1r* (**i**), *Satb2* (**j**), *Aox3* (**k**), and *Brs3* (**l**) from intermediate to caudal PVH at representative bregma levels (−0.7mm to −1.0mm).

PVH regulation of feeding behavior has been studied extensively, yet the precise PVH neurons mediating satiety are still unknown. Further, several marker genes expressed by PVH neurons have been proposed to be involved in satiety regulation, but the relationship among these genes is unresolved. Given the limited number of centrally-projecting PVH neurons and the low expression of many satiety-associated genes, prior studies have lacked the sample size and/or sensitivity to reliably characterize the expression of satiety genes in different PVH neuron populations. Therefore, since MERFISH has increased sensitivity over droplet-based sc/snRNA-seq methods^48^, we examined our *Sim1*^+^ MERFISH atlas to assess the expression patterns of genes associated with satiety. To begin, we analyzed expression of *Mc4r* as MC4R signaling in the PVH is necessary and sufficient for satiety and body weight regulation^12–15,21^. *Mc4r* is expressed by several *Sim1*^+^ neuron populations and highly correlated with expression of *Npy1r*, as expected, given its role in feeding behavior (Fig. 6a–c)^92–94^. Expression of *Mc4r* and *Npy1r* is widespread throughout the PVH, with an enrichment in the Caudal-Intermediate region between bregma levels −0.7 mm to −1.0 mm (Fig. 6h,i). Despite *Mc4r* being expressed by multiple PVH neuron subtypes, three clusters display the strongest enrichment, MF_S2.Trh-Satb2, MF_S11.Aox3, and MF_S14.Brs3 (Fig. 6a,d–g). These marker genes, *Satb2*, *Aox3*, and *Brs3*, have limited spatial distributions, often enriched within areas of high *Mc4r* and *Npy1r* expression (Fig. 6j–l). MF_S2.Trh-Satb2 neurons have the highest expression of *Mc4r* and represent PVH^*Trh*^ neurons that project to the median eminence (Fig. 1c,d,f) to control the hypothalamic-pituitary-thyroid axis, which is consistent with MC4R and NPYregulation of thyroid hormone release during fasting^95^. The next highest *Mc4r*-expressing clusters are MF_S11.Aox3 and MF_S14.Brs3, both of which project centrally as they are not labeled by systemic Fluoro-Gold injection (**Extended Data Fig. 10a,b**). MF_S11.Aox3 represents a novel population of centrally-projecting PVH neurons with unknown function(s), while PVH^*Brs3*^ neurons regulate feeding behavior, as noted above^67^. In support of an interaction between *Brs3* and *Mc4r*, conditional knockout of *Brs3* from *Mc4r*-expressing neurons produces obesity^96^.

Other genes used to investigate PVH satiety-regulating populations, including *Calcr*^16^, *Glp1r*^15^, *Irs4*^17^, *Ntrk2*^18^, *Nos1*^19^, *and Pdyn*^21^, are expressed widely across different PVH neuron subtypes (**Extended Data Fig. 10c)**. Among them, *Calcr* and *Glp1r* have the most restricted expression patterns but are expressed by neuroendocrine and centrally-projecting populations. With regard to identifying candidate PVH satiety neurons within our atlas, three clusters express the majority of the satiety genes above (i.e., *Calcr*, *Glp1r*, *Irs4*, *Ntrk2*, *Nos1*, and *Pdyn*), MF_S8.Esr2-Ret, MF_S13.Pde3a-Tmem215, and MF_S14.Brs3 (**Extended Data Fig. 10c**). As noted before, MF_S14.Brs3 neurons are enriched for *Mc4r* expression and may represent *Mc4r*-expressing satiety neurons. MF_S8.Esr2-Ret and MF_S13.Pde3a-Tmem215 neurons, on the other hand, express little *Mc4r* but co-express *Glp1r* and *Pdyn* (**Extended Data Fig. 10c-g**). Given that PVH^*Pdyn*^ and PVH^*Glp1r*^ neurons are key regulators of satiety and body weight^15,21,97^, and PVH^*Mc4r*^ and PVH^*Pdyn*^ neurons are distinct satiety-regulating populations^21^, MF_S8.Esr2-Ret and MF_S13.Pde3a-Tmem215 neurons are candidates to be the *Pdyn*-expressing PVH satiety neurons.

### Targeted transcriptomic profiling of PVH *Sim1*^+^ neurons that project to the parabrachial region.

**Figure 7:**
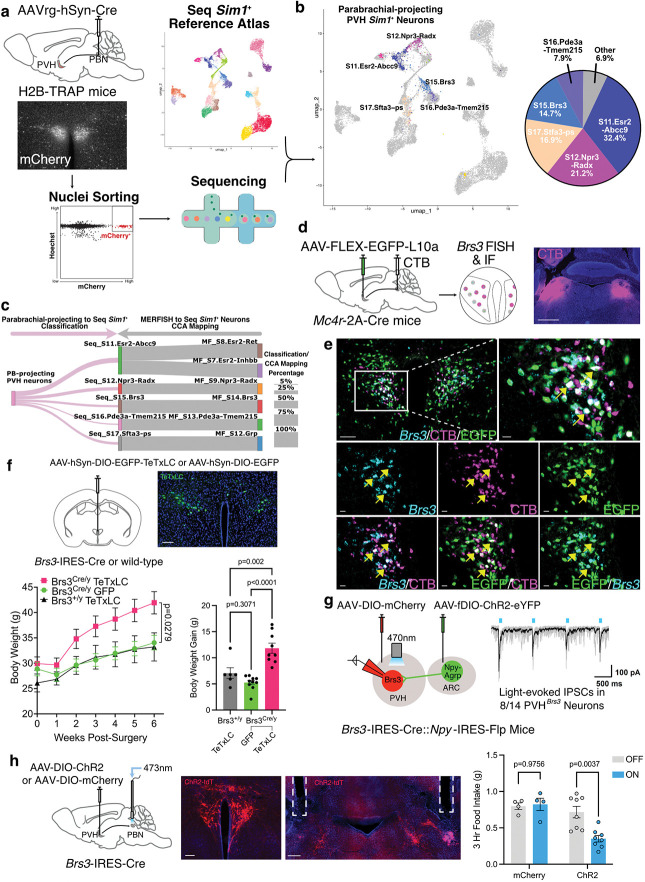
PVH^Brs3^ neurons project to the parabrachial region and promote satiety **a**) Experimental workflow for targeted single-nucleus RNA sequencing of parabrachial (PB)-projecting PVH neurons. **b**) PB-projecting PVH neurons were classified with the *Sim1*^+^ sc/snRNA-seq reference atlas and projected into its UMAP space. The pie chart illustrates the proportion of PB-projecting PVH neurons that map to individual *Sim1*^+^ neuron clusters. **c**) Three-level Sankey plot showing the mapping percentage of PB-projecting PVH neurons classified using the mouse *Sim1*^+^ sc/snRNA-seq reference atlas (left mapping to center; lower 5% not shown) with corresponding *Sim1*^+^ MERFISH clusters identified via canonical correlation analysis (CCA; right mapping to center). Line thickness represents strength of mapping. **d**) Schematic diagram of retrograde cholera toxin subunit B (CTB) injection into the PB and Cre-dependent AAV-EGFP-L10a injection into the PVH of a *Mc4r*-2A-Cre mouse (left). Representative coronal brain section showing the bilateral PBN CTB injection sites labeled by CTB IF. Scale bar = 250 μm (right). **e**) Top panels show low magnification (left) and high magnification (right) views of EGFP immunofluorescence (IF), CTB IF, and *Brs3* fluorescence in situ hybridization (FISH) labeling. Middle panels display *Brs3* FISH, CTB IF, and EGFP IF, respectively, from left to right. Bottom panels display the overlay of *Brs3* FISH with CTB IF (left), EGFP IF with CTB IF (center), and EGFP IF and *Brs3* FISH (right). Yellow arrows indicate representative triple-labeled neurons. Low magnification scale bar = 100 μm, high magnification scale bar = 20 μm. **f)** Schematic of Cre-dependent AAV-tetanus toxin light chain (TeTxLC) or AAV-EGFP injection into the PVH of *Brs3*-IRES-Cre or wild-type mice (Top left). Representative PVH injection site labeled with Cre-dependent AAV-EGFP-TeTxLC. Scale bar = 250 μm (Top right). Experimental groups include wild-type (Cre-negative) mice injected with Cre-dependent AAV-TeTxLC (n = 6), *Brs3*-IRES-Cre mice injected with Cre-dependent AAV-GFP (n = 10), and *Brs3*-IRES-Cre mice injected with Cre-dependent TeTxLC (n = 9). Body weights were monitored from the day of surgery (week 0) (lower left), and total body weight gained over 6 weeks post-surgery is depicted (lower right). Statistical analysis was performed using one-way ANOVA followed by Tukey’s multiple comparisons test (** *P* < 0.01, **** *P* < 0.0001). **g**) Schematic and representative trace from Arc^*Npy/Agrp*^ → PVH^*Brs*3^ neuron channelrhodopsin-2 (ChR2)-assisted circuit mapping (CRACM) experiment. Light-evoked IPSCs were detected in 8/14PVH^*Brs*3^ neurons. **h**) Schematic showing injection of Cre-dependent ChR2 or mCherry into the PVH and optic fiber implants over the PB in *Brs3*-IRES-Cre mice with representative brain sections showing bilateral ChR2 expression in the PVH (scale bar = 100 μm), and bilateral fiber tracks in the PB (scale bar = 250 μm;right). Food intake (right) was measured over the first 3-hours of the dark-cycle, with or without photostimulation, in mCherry- (n = 4) and ChR2-expressing mice (n = 8). Statistical analysis was performed using two-way repeated measures ANOVA followed by Sidak’s multiple comparisons test (** *P* < 0.01).

PVH neurons promote satiety through direct excitatory projections to the PB. PVH^*Mc4r*^ neurons elicit robust glutamatergic synaptic responses in downstream neurons located in the lateral parabrachial nucleus (LPBN)^12^, whereas PVH^*Pdyn*^ neurons preferentially do so in neurons found in the nearby pre-locus coeruleus (pLC)^21,98^, despite each satiety population projecting to both regions. That said, *Mc4r* and *Pdyn* are expressed by multiple PVH neuron subtypes, as noted above, and specific molecular markers for PB-projecting PVH neurons have not been identified. Hence, the precise PVH neurons that regulate satiety are unknown. To elucidate the specific PVH populations that project to the PB, we performed targeted snRNA-seq similar to spinal cord-projecting neuron profiling above. Retrograde Cre virus was injected bilaterally into the PB, targeting the LPBN and adjacent pLC, to selectively label the nuclei of PB-projecting PVH neurons with mCherry. Next, PB-projecting nuclei were isolated, collected via FANS, and sequenced (Fig. 7a). After clustering, PB-projecting *Sim1*^+^ neurons were classified based on our *Sim1*^+^ PVH sc/snRNA-seq atlas and projected onto the reference UMAP (Fig. 7b, **Supplementary Table 30**). Our results show that most of the PB-projecting PVH neurons cluster with one of the following populations: Seq_S11.Esr2-Abcc9 (32.4%), Seq_S12.Npr3-Radx (21.2%), Seq_S15.Brs3 (14.7%), Seq_S16.Pde3a-Tmem215 (7.9%), or Seq_S17.Sfta3-ps (16.9%). Of interest, *Mc4r-* and *Npy1r*-enriched MF_S14.Brs3 neurons correspond to the Seq_S7.Brs3 cluster based on our *Sim1*^+^ MERFISH to sc/snRNA-seq CCA mapping (Fig. 7c). To confirm that PVH^*Brs3*^ neurons express *Mc4r* and project to the PB, we injected the retrograde tracer cholera toxin subunit B (CTB) into the PB and Cre-dependent AAV-EGFP-L10a into the PVH of *Mc4r*-2A-Cre mice^12,99^. Subsequently, we performed FISH to detect *Brs3* expression in the PVH. Histological analysis revealed triple-labeling of fluorescent signals from *Brs3* FISH, *Mc4r*-positive neurons labeled with EGFP, and PB-projecting PVH neurons labeled with CTB (Fig. 7d,e). Collectively, these findings support the hypothesis that PVH^*Brs3*^ neurons regulate satiety.

### PVH^*Brs3*^ neurons regulate feeding via projections to the PB.

Given the importance of PVH^*Mc4r*^ neurons to energy balance and prior studies demonstrating PVH^*Brs3*^ neuron inhibition increases food intake^67^, we next asked whether PVH^*Brs3*^ neurons are necessary for body weight regulation. To test this, we silenced PVH^*Brs3*^ neurons by bilaterally injecting an AAV driving Cre-dependent expression of tetanus toxin light chain (TeTxLC) or GFP as control into the PVH of *Brs3*-IRES-Cre mice^100^. Additionally, we injected a cohort of wild-type mice with Cre-dependent AAV-TeTxLC as another control group. Body weights were measured weekly, and after six weeks, *Brs3*-IRES-Cre mice receiving TeTxLC gained significantly more body weight compared to both control groups (Fig. 7f). This finding demonstrates that PVH^*Brs3*^ neurons regulate body weight by preventing weight gain.

PVH^*Mc4r*^ neurons are directly inhibited by ARC^*Agrp*^ neurons to induce hunger^12,21^. Since PVH^*Brs3*^ neurons express *Mc4r*, project to the PB, and have been implicated in feeding behavior regulation, we next tested whether they receive synaptic input from ARC^*Agrp*^ neurons^12,21^. ARC^*Agrp*^ à PVH^*Brs3*^ neuron connectivity was assessed by channelrhodopsin-2 (ChR2)-assisted circuit mapping (CRACM) using *Brs3*-IRES-Cre::*Npy*-IRES-Flp^101^ mice as *Npy* and *Agrp* are co-expressed in the ARC^102^. Cre-dependent AAV-mCherry was injected into the PVH to visualize *Brs3*-expressing neurons for *ex vivo* brain slice electrophysiology recordings, and Flp-dependent AAV-ChR2-eYFP was injected into the ARC to drive ChR2 expression in NPY/AgRP neurons. Light-evoked inhibitory postsynaptic currents (IPSCs) were detected in 8 out of 14 PVH^*Brs3*^ neuron recordings (Fig. 7g), indicating ARC^*Agrp*^ neurons are monosynaptically connected to many PVH^*Brs3*^ neurons – further supporting their role in satiety regulation. Having established that PVH^*Brs3*^ neurons receive input from ARC^*Agrp*^ neurons, we next asked if PVH^*Brs3*^ projections to the PB are sufficient to reduce food intake using *in vivo* optogenetics. *Brs3*-IRES-Cre mice were injected with either Cre-dependent AAV-ChR2 or AAV-mCherry into the PVH, and optical fibers were implanted bilaterally above the PB. Photostimulation of ChR2-expressing PVH^*Brs3*^ à PB terminals at the onset of the dark cycle significantly reduced food intake (Fig. 7h), which is consistent with the effects observed after chemogenetic activation of the entire PVH^*Brs3*^ population^67^ and photostimulation of PVH^*Mc4r*^ neuron projections to the PB^12^. No reduction in food intake was observed in the mCherry control group after photostimulation. Together, these data establish PVH^*Brs3*^ neurons as a precise neuronal subtype mediating satiety via projections to the PB.

## Discussion

We leveraged single-cell and spatial transcriptomics technologies to develop a high-resolution, spatially resolved atlas of the mouse PVH region. Extensive transcriptional profiling enabled a detailed analysis of the molecular diversity among PVH cell types, highlighting stark differences between neuroendocrine and centrally-projecting PVH neurons. Using the marker gene profiles revealed by sc/snRNA-seq, we then performed MERFISH on the PVH region from multiple male and female mice, yielding spatial transcriptomic information from 41 coronal sections spanning bregma levels −0.4 mm to −1.2 mm, and including more than 150,000 cells. Specific analysis of *Sim1*^*+*^ neurons identified by MERFISH revealed 26 transcriptionally distinct populations, six of which were neuroendocrine, expressing peptide hormones along with secondary markers that exhibited highly specific expression patterns. Spatial domain analysis of MERFISH data further designated *Sim1*^*+*^ neurons as PVH or Peri-PVH, and segregated PVH neurons into Rostral, Rostral-Intermediate, Caudal-Intermediate, or Caudal groups. Analysis of the similarity between *Sim1*^*+*^ neuron populations identified by MERFISH and sc/snRNA-seq demonstrated remarkably high correspondence for neuron subtypes located within the PVH. Our study also cataloged 29 GABAergic neuron subtypes that surround the PVH, highlighting the significant heterogeneity of this region and providing a means for gaining selective genetic access to these neuron populations for future investigation.

Noted above, our atlas provides molecular markers capable of distinguishing neuroendocrine and centrally-projecting PVH neurons that express the same neuropeptide hormone gene, which is of great interest for PVH^*Crh*^ and PVH^*Oxt*^ neurons that control stress-related and social behaviors^103–105^. For instance, PVH^*Crh*^ neuroendocrine neurons (Seq_S1.Crh-Scgn) express *Scgn*, whereas centrally-projectingPVH neurons expressing *Crh* include Seq_S12.Npr3-Radx, Seq_S13.Npy2r-Tll2, Seq_S14_Aox3, and Seq_S15_Brs3 clusters, all of which lack *Scgn* expression. These cluster-specific genetic markers make it possible for future functional studies to selectively target PVH^*Crh*^ and PVH^*Oxt*^ neuron subtypes and link them to distinct physiological and behavioral phenotypes. However, it remains unclear which centrally-mediated behaviors are driven by collateral projections from neuroendocrine populations to other hypothalamic sites^106–109^ versus those resulting from distinct centrally-projecting PVH^*Crh*^ and PVH^*Oxt*^ neurons.

In addition to our spatially resolved atlas, we conducted targeted snRNA-seq of spinal cord- and PB-projecting PVH neurons to elucidate the neuronal populations involved in regulating sympathetic nervous system activity and feeding behavior, respectively. Prior studies have identified several marker genes for spinal cord-projecting PVH neurons, including *Avp*, *Oxt*^4,8,110–112^, *Bdnf* ^23^, *Mc4r*^12^, *Nos1*, *Sim1*^19^, *Erbb4*, *Otp*, *Pcsk5*, *Prlr*, and *Zeb2*^89^, but none of these genes are unique to a single PVH neuron type. Further, prior sc/snRNA-seq studies only profiled cervical- and lumbar-projecting neurons in the brain^90^. Given our interest in the regulation of the sympathetic nervous system, we sequenced PVH neurons that project to the thoracic cord, where preganglionic neurons are primarily located. Our results for thoracic-projecting neurons aligned well with publicly available data as all spinal cord-projecting PVH neurons predominantly mapped to Seq_S11.Esr2-Abcc9, Seq_S12.Npr3-Radx, and Seq_S10.Npsr1-Npnt clusters of our *Sim1*^+^ sc/snRNA-seq reference atlas. The functional roles of these neuron populations remain unknown, but pharmacological manipulation of ESR2 and NPR3 activity in the PVH has been shown to reduce blood pressure^62,63,65^. Of interest, it has long been recognized that a small number of PVH^*Avp*^ and PVH^*Oxt*^ neurons project to the spinal cord; however, none of the sequenced spinal cord-projecting PVH neurons mapped to neuroendocrine Seq_S5.Oxt-Rxfp3 or Seq_S6.Avp-Pla2r1 clusters. This is consistent with neuroanatomical tracing studies showing pituitary-projecting PVH neurons do not collateralize to the brainstem and spinal cord^1,5^. Therefore, spinal cord-projecting PVH^*Avp*^ and PVH^*Oxt*^ neurons likely belong to the centrally-projecting Seq_S11.Esr2-Abcc9 population, which is positive for both *Avp* and *Oxt*.

We sequenced PB-projecting PVH neurons to ascertain their cell type identities because PVH^*Mc4r*^ and PVH^*Pdyn*^ satiety neuronsrepresent distinct PB-projecting populations, andmultiple neuron subtypes express *Mc4r* and *Pdyn*^12,21^. The majority of *Sim1*^+^ PB-projecting neurons mapped to five clusters, including two spinal cord-projecting sc/snRNA-seq clusters, Seq_S11.Esr2-Abcc9 and Seq_S12.Npr3-Radx. This may represent similarities in transcriptomes between PB- and spinal cord-projecting PVH neurons or that some PVH neurons collateralize between these two regions. However, we cannot rule out that retrograde AAV injections were taken up by spinal cord-projecting fibers passing through the PB, which has been observed with some retrograde tracers^113^. PB-projecting neurons did map to three clusters that spinal cord-projecting PVH neurons did not, including Seq_S15.Brs3, Seq_S16.Pde3a-Tmem215, and Seq_S17.Sfta3-ps. Taking advantage of the enhanced gene detection capability of MERFISH, we were able to identify the PVH neurons with the highest expression of *Mc4r* and compare them with those identified as PB-projecting. Notably, the MF_S14.Brs3 cluster is among the highest expressors of *Mc4r* and corresponds to the PB-projecting cluster Seq_S15.Brs3. This information, in conjunction with prior work demonstrating that chemogenetic activation of PVH^*Brs3*^ neurons reduces food intake and inhibition does the opposite^67^, inspired us to further examine their role in energy balance. We show that 1) chronic PVH^*Brs3*^ neuron silencing causes significant weight gain, 2) they receive direct GABAergic input from hunger-driving ARC^*Agrp*^ neurons, and 3) stimulation of PVH^*Brs3*^ neuron projections to the PB reduces food intake. These results are all consistent with PVH^*Brs3*^ neurons representing *Mc4r*^+^ satiety neurons, yet the effects on food intake that we and others observed were smaller compared to manipulating all PVH^*Mc4r*^ neurons^12,67^. Thus, there may be multiple PVH^*Mc4r*^ neuron populations that control food intake. With regard to pinpointing the specific cluster containing PVH^*Pdyn*^ satiety neurons^21^, MF_S8.Esr2-Ret and MF_S13.Pde3a-Tmem215 neurons correspond to PB-projecting PVH neurons that express *Pdyn* and *Glp1r* but lack *Mc4r*. However, additional studies are required to test whether PB-projecting MF_S8.Esr2-Ret and/or MF_S13.Pde3a-Tmem215 neurons control satiety.

This atlas of the PVH serves as a versatile resource to support future studies of PVH organization and function. It also has several advantages over prior work^25,26^, including a vastly increased sample size, both unbiased and circuit-based molecular profiling, and the ability to resolve spatial information with MERFISH using a gene panel curated for the PVH and surrounding regions. To facilitate accessibility for the scientific community, we uploaded our analyzed sc/snRNA-seq and MERFISH data to the Broad Single Cell Portal (https://singlecell.broadinstitute.org/single_cell/study/SCP2858), an open-access, web-based tool for exploring single-cell genomics data – thus, providing a valuable resource for the field of homeostasis.

## Methods

### Mice:

All animal care and experimental procedures were approved by the Institutional Animal Care and Use Committees at Beth Israel Deaconess Medical Center and the University of Iowa. Prior to the start of experiments, mice were housed in a temperature- and humidity-controlled room with a 12-hr light-dark cycle and maintained on standard diet (Inotiv 7913) unless stated otherwise. C57BL/6J background wild-type mice were used for the majority of single-cell and single-nucleus RNA sequencing experiments as well as MERFISH experiments. In some cases, *Sim1*-Cre (JAX006395^14^), *Sim1*-Cre::R26-LSL-EGFP-L10a^22^, or H2B-TRAP mice (JAX029789^91^) were used for single-cell and single-nucleus RNA sequencing studies to guide dissections and sample collection with FANS. Behavior experiments were completed with *Brs3*-IRES-Cre mice (JAX030540^100^), which were crossed to *Npy*-IRES-Flp (JAX030211^101^) mice for CRACM experiments. Additionally, *Slc17a6*-IRES-Cre (JAX028863^114,115^)::R26-LSL-EGFP-L10a^22^, *Slc32a1*-IRES-Cre (JAX028862^114^)::R26-LSL-EGFP-L10a, *Mc4r*-2a-Cre (JAX030759^12^), and C57BL/6J wild-type mice were used for histological experiments.

### Single-cell/nucleus RNA sequencing tissue collection, library preparation, and sequencing:

C57BL/6J, *Sim1*-Cre, or *Sim1*-Cre::R26-LSL-EGFP-L10a mice aged 6–12 weeks were sacrificed between 9 am – 12 pm by rapid decapitation immediately after removal from the home cage. Brains were extracted and chilled in DMEM/F12 media slush. Next, brains were placed ventral side up in a chilled stainless steel brain matrix (Roboz Surgical Instrument Co.: SA-2165), and 1 mm coronal sections of the hypothalamus were collected. The PVH was then micro-dissected under a fluorescent stereoscope. For each sample preparation, 4–10 male or female mice were pooled. Sample and library preparation was performedas described previously for Drop-seq^116^ and DroNc-seq^46^ with minor modifications. One of three DroNc-seq samples was prepared from fasted C57BL/6J mice. For 10X Chromium v3 sequencing runs, samples and library preps were prepared as described previously for incorporation with fluorescence-activated nuclei sorting (FANS) with minor modifications^117,118^. In addition, subsets of 10X Chromium v3 samples from *Sim1*-Cre mice injected with AAVDJ-hSyn-H2B-mCherry (Boston Children’s Hospital Viral Core)^119^ or H2B-TRAP mice injected with AAVrg-hSyn-Cre, for projection-specific snRNA-seq experiments described below, were incubated with hashtag oligos for 15 minutes for eventual multiplexing prior to FANS enrichment based on nuclear mCherry. Multiplexed *Sim1*-Cre samples were obtained from mice that were *ad libitum* fed, fasted, or refed for 60 minutes before sacrifice. Libraries were sequenced on an Illumina NextSeq 500 or Illumina NovaSeq 6000 at a minimum read depth of 20,000 reads per cell/nucleus. Hashtag oligo libraries were sequenced to a minimum read depth of either 1,000 or 5,000 reads/nucleus and processed into count matrices using either the Cumulus Tool on Feature Barcoding (https://github.com/lilab-bcb/cumulus_feature_barcoding) or kallisto | bustools (https://www.kallistobus.tools/). For Drop-seq and DroNc-seq data, raw sequencing reads were processed using the Drop-seq tools pipeline^46,116^. Barcodes with base quality <10 were removed, and 5’ and 3’ ends of reads were trimmed to remove TSO and poly(A) tails, respectively. Reads were then aligned to the GRCm38 reference genome using STAR v2.7.7. Feature-barcode matrices were then generated by summing detected unique molecular identifiers (UMIs) for each barcode with errors corrected at a hamming distance of 1. For 10X Chromium v3 libraries, 10X Genomics Cell Ranger was used to map reads to the GRCm38 reference genome and generate feature-barcode matrices.

### Single-cell/nucleus RNA sequencing Quality Control:

For all sequencing data regardless of technology, CellBender (v0.2.2) was used to identify and filter out reads captured from ambient RNA and random barcode swapping^120^. Subsequently, data from Drop-seq, DroNc-seq, and 10X Chromium v3 sequencing runs were loaded into an RStudio environment (R v 4.4.1) and processed through a custom Seurat-based analysis pipeline run in Seurat v5.0.1.9001^35^. First, we applied additional filtering to remove cells/nuclei with fewer than 250 unique genes. DroNc-seq data were then filtered to exclude nuclei with total UMI count outside the range of 1,000 to 10,000, while Drop-seq and 10X chromium-v3 data were filtered to exclude cells/nuclei with total UMI count outside the range of 1,000 to 25,000. Additionally, *PercentFeatureSet*() was used to calculate mitochondrial gene expression, and cells/nuclei from all datasets were removed if they had a mitochondrial gene expression rate of greater than 10%. Finally, all cells/nuclei with a ratio of log_10_(unique genes)/log_10_(unique molecules) less than 0.8 were removed. After quality control filtering was complete, all data were merged into a single Seurat object for integrated analysis.

### Single-cell/nucleus RNA sequencing and data integration:

For integrated analysis, 11 batches of sequencing runs were merged into a single Seurat object (Drop-seq = 7 batches, DroNc-seq = 2 batches, and 10X Chromium-v3 = 2 batches) followed by joining of the “RNA” assay layers using JoinLayers(). Raw counts were log-normalized, using Seurat *NormalizeData()*, and cell cycle scoring for S phase and G2/M was computed using the Seurat *CellCycleScoring()* function^121^. Subsequently, given that stress readily activates PVH neurons, particularly PVH^*Crh*^ neurons controlling the HPA axis, the *AddModuleScore()* function was used to measure the expression level of a set of 19 primary rapidly responding activity-dependent genes to compute a “cellular activation score” based on this transcriptional signature for each cell^122^. Next, layers were split by sequencing run (“batch”), and *FindVariableFeatures()* was used to select the top 5,000 highly variable genes. Data were then scaled with *ScaleData()*, while regressing out the following covariates: mitochondrial gene percentage, cell-cycle scores, and cellular activation score. Principal component analysis (PCA) was performed with the *RunPCA*() function. Following calculation of principal components, integration of layers was carried out using *IntegrateLayers()* with reciprocal principal component analysis (RPCA)-based integration^36^. After integration, we used the top 30 principal components for clustering and dimensionality reduction using the Seurat *FindNeighbors()*, *FindClusters(),* and *RunUMAP()* functions. To identify marker genes for each cluster, we re-joined layers using *JoinLayers()* and ran *FindAllMarkers()* for differential gene expression analysis (DGEA) using the non-parametric *Wilcoxon Rank Sum test*. Differentially expressed genes were defined as those with > 0.2 average log_2_ fold change and a *Bonferroni-corrected* p-value less than 0.01. Marker gene analysis guided identification of doublets/multiplets, which were classified as clusters that expressed high levels of more than one canonical cell type marker genes (e.g., clusters expressing marker genes for both neurons and astrocytes) and were removed. In addition, clusters comprised of doublets/multiplets and/or “low quality” metrics, including mitochondrial gene enrichment or absence of cell type-defining markers indicating low complexity, were removed. This process was repeated at several levels of analysis, beginning with all cells, then after subclustering for neurons, GABAergic neurons, glutamatergic neurons, and *Sim1*-expressing neurons.

### Integration of Sim1-expressing clusters with publicly available HypoMap and Allen Brain Cell Atlas data:

To integrate *Sim1*^+^ PVH sc/snRNA-seq data from our study with publicly available sequencing data from the murine PVH, we downloaded data from HypoMap, an integrated atlas of mouse hypothalamus^32^. Using the provided anatomical annotations with the Seurat object, we subset for and clustered only cells/nuclei annotated as “paraventricular hypothalamic nucleus” using the pipeline described for this study. Notably, during clustering, we curated the HypoMap data for *Sim1*-expressing cells/nuclei, filtering out any clusters marked by specific expression of GABAergic or thalamic marker genes (i.e., *Slc32a1* and *Tcf7l2*). We then merged and integrated the HypoMap PVH *Sim1*^+^ neurons with PVH *Sim1*^+^ sc/snRNA-seq data collected in this study using our Seurat-based analysis workflow. However, after integration, inconsistencies were observed across datasets. We then instead integrated publicly available PVH *Sim1*^+^ scRNA-seq data from the whole mouse brain Allen Brain Cell (ABC) Atlas with *Sim1*^+^ PVH sc/snRNA-seq data from this study^29^. To specifically access PVH cells from the ABC Atlas, we first downloaded two H5 AnnData expression matrices (WMB-10Xv2-Hy-raw.h5ad and WMB-10Xv3-Hy-raw.h5ad) containing all cells collected from hypothalamic dissections and sequenced using either 10X Chromium v2 or 10X Chromium v3 chemistry. Subsequently, we used the *Convert()* and *LoadH5Seurat()* functions toload the ABC Atlas data into a Seurat object and used the published taxonomic classifications to select for data from the PVH region. The ABC Atlas assigned anatomical annotation was used to specifically select clusters that spatially mapped to either the PVH (“PVH”) or the anterior portion of the periventricular area (“PVa”). Subsequently, we further filtered our selection only to keep glutamatergic clusters using the ABC Atlas assigned neurotransmitter type label, keeping clusters annotated as either “Glut” or “Glut-GABA”. We then removed cells with a mitochondrial gene expression rate greater than 10% and clustered the data in Seurat version 5. For clustering, ABC Atlas data were processed as described above with minor modifications. Notably, the 10X chemistry (i.e., v2 and v3) were each treated as a “batch” for integrated analysis. After clustering, any identified “low quality” or doublet/multiplet clusters were removed as described above. Finally, we merged and integrated the ABC Atlas *Sim1*^+^ neurons with the PVH *Sim1*^+^ sc/snRNA-seq data from this study following the workflow described above.

### Analysis of neuroendocrine neuron transcriptional profiles:

DGEA was run using Seurat *FindMarkers()* on the different neuron classes: centrally-projecting, neuroendocrine, median eminence-projecting, and posterior pituitary-projecting neurons. Differentially expressed genes were defined as having > 0.2 average log_2_ fold change and a *Bonferroni-corrected* p-value <0.01. Next, the clusterProfiler package was used to perform Gene Ontology (GO) enrichment analysis of genes differentially expressed by centrally-projecting, neuroendocrine, median eminence-projecting, and posterior pituitary-projecting neuronal classes^123^. Specifically, *compareCluster()* was used to perform “enrichGO” analysis, which executes an over-representation analysis^124^ for all GO ontology categories (i.e., biological process, cellular component, and molecular function) with *Bonferroni correction* for multiple comparisons at an alpha value of 0.05.

### Single-nucleus RNA sequencing of projection-specific PVH neuron populations:

We sequenced projection-specific PVH neurons using either 10X Chromium v3 or Smart-Seq2 (“sNuc-seq”)^125,126^ technologies. For 10X Chromium v3 experiments, H2B-TRAP mice received bilateral stereotaxic injections of AAVrg-hSyn-Cre (Addgene #105553) into either the upper thoracic spinal cord or the parabrachial region. sNuc-seq samples were prepared by bilaterally injecting C57BL/6J mice with AAVDJ-hSyn-H2B-mCherry into the PVH and AAVrg-CAG-GFP-Cre (Boston Children’s Hospital Viral Core) or HSV-hEf1a-mCherry-IRES-Cre (Mass General Brigham Gene Delivery Technology Core; Dr. Rachael Neve) into the PB. Two weeks post-surgery, animals were sacrificed, and tissue was collected as described above. Samples processed with 10X Chromium v3 were completed as described in Schwalbe *et al.*^117^, while sNuc-seq was performed as described in Tao *et al.*^119^. Spinal cord-projecting data consists of two 10X Chromium v3 sequencing runs, while the parabrachial-projecting data consists of two runs of 10X Chromium v3 and two sNuc-Seq sequencing runs. In addition, we downloaded two publicly available spinal cord-projecting datasets (GEO accession numbers GSE247594 and GSE212409)^89,90^ and accordingly classified these data using our *Sim1*^+^ PVH sc/snRNA-seq reference atlas. Briefly, we calculated the percentage of mitochondrial gene expression using Seurat’s *PercentFeatureSet*() to identify and remove any cells/nuclei with a mitochondrial gene expression rate greater >10%. Cells/nuclei with fewer than 1000 UMIs were also removed from further analysis. Subsequently, we clustered all parabrachial- and spinal cord-projecting data using the analysis pipeline described above and filtered the data to only retain *Sim1*-expressing clusters. To classify each cell, we proceeded to use *FindTransferAnchors*() to project our mouse *Sim1*^+^ sc/snRNA-seq reference atlas PCA structure onto the parabrachial- and spinal cord-projecting data to identify paired anchor cells across datasets. We then used the identified anchors and the *MapQuery*() function to map parabrachial- and spinal cord-projecting data into our mouse *Sim1*^+^ sc/snRNA-seq reference atlas UMAP space.

### Analysis of human PVH single-nucleus RNA sequencing data:

Two published datasets contain snRNA-seq data from the hypothalamus of adult humans^75,76^. From Siletti et al., 2023^75^, we downloaded a Seurat object containing data from dissections encompassing the medial preoptic region of the hypothalamus, supraoptic region of the hypothalamus, and paraventricular nucleus of the hypothalamus. We then filtered the data for neurons with > 1,000 UMIs and < 10% mitochondrial gene expression and retained *SIM1*^+^ clusters for further analysis. After filtering for *SIM1*^+^ neurons, the data included samples from one 60-year-old female and one 50-year-old male. We also downloaded a Seurat object from Tadross et al., 2025^76^, containing data from the entire adult human hypothalamus, which was filtered as above and contributed data from two females, aged 63 and 94 years, and four males, aged 83, 88, 91, and 94. After analyzing the integrated human *SIM1*^+^ data, we used a text file (“gene_ortologs.gz”) available from NCBI (https://ftp.ncbi.nlm.nih.gov/gene/DATA/) to identify all gene homologs present in both the human *SIM1*^+^ object and our mouse *Sim1*^+^ sc/snRNA-seq atlas. We then completed a canonical correlation analysis (CCA) to assess the transcriptional similarity of each cluster between the human and mouse atlases by using Seurat’s *FindTransferAnchors*() and *TransferData*() functions.

### MERFISH gene panel selection:

A gene panel of 503 genes (**Supplementary Table 16**) was curated specifically for the PVH and surrounding regions based on differentially expressed genes identified in sc/snRNA-seq experiments (**Supplementary Tables 1–4,7**), canonical marker genes for neurons and non-neuronal cells, and functionally important genes described in the scientific literature. After gene selection, Vizgen manufactured the custom “MERFISH 500 Gene Panel” (Vizgen: 20300008), comprised of probes targeting a minimum of 30 regions per gene (except for *Avp* and *Oxt*) and using a 25-bit binary code readout for gene assignment after combinatorial single molecule FISH (smFISH). Furthermore, 50 “blanks” comprising non-encoding scrambled sequences were included in the gene panel as negative controls (**Supplementary Table 17**). Three of the 503 genes, *Avp*, *Oxt*, and *Sst*, were assigned to the “sequential panel” to avoid optical overcrowding artifacts due to high abundance of expression. Genes in the sequential panel are detected using unique probes identified by their direct fluorescent signal in distinct imaging rounds occurring after combinatorial smFISH imaging.

### MERFISH tissue collection and sample preparation:

MERFISH experiments were conducted according to Vizgen MERSCOPE protocols for fresh frozen tissue using six C57BL/6J mice, comprised of four males and two females, aged 8–10 weeks. Sacrifice and brain extraction was done as described for sc/snRNA-seq studies above. Brains were then positioned ventral side up in a chilled stainless steel brain matrix and sliced into 3-mm thick coronal slices that included the PVH region. Subsequently, the coronal slices were placed anterior side up and trimmed dorsally, removing tissue above the lateral septum, and laterally to remove cortex and much of the striatum. PVH tissue blocks were then embedded in a square mold (S22, Kisker Biotech) with Tissue-Tek^®^ O.C.T. Compound (Sakura) and stored at −80°C until sectioning. Tissue blocks were placed in a cryostat (Epredia CryoStar NX50 HD Cryostat) and incubated at −20°C for 1 hour prior to sectioning coronally at 10 μm thickness. We mounted 4–10 sections from each brain at ~100 μm intervals onto warm MERSCOPE slides (Vizgen: 20400001), beginning at approximately bregma level −0.4 mm and continuing to −1.2 mm according to the Franklin-Paxinos atlas^49^. After sectioning, MERFISH slides were placed face-up in a 60 mm petri dish (VWR, 25382–687) and left at room temperature for 5 minutes. Next, slides were incubated in freshly made 4% paraformaldehyde (PFA; Electron Microscopy Sciences: 15714-S) in RNase-free phosphate-buffered saline (PBS; Thermo Fisher Scientific: AM9625) for 15 minutes at room temperature. Slides were then washed three times for five minutes each with PBS at room temperature and treated with freshly made 70% ethanol for tissue permeabilization and storage for a minimum of 24 hours at 4°C in parafilm-sealed 60 mm dishes.

### MERFISH probe hybridization and imaging:

Slides were taken out of 4°C and washed with Sample Preparation Wash Buffer (Vizgen: 20300001) for five minutes at room temperature, followed by incubation in Formamide Wash Buffer (Vizgen: 20300002) for 30 minutes at 37°C. Subsequently, our custom 503 gene MERSCOPE panel for the PVH was applied to the slides with a parafilm coverslip and incubated at 37°C for 36–42 hours. Slides were then washed twice with Formamide Wash Buffer for 30 minutes each at 47°C. To gel-embed tissue samples on slides, a mix composed of Gel Embedding Premix (Vizgen: 20300004), ammonium persulfate (APS; Sigma: 09913–100G), and TEMED (Sigma: T7024–25ML) was prepared and applied to the tissue. A circular Gel Coverslip (Vizgen: 30200004), treated with RNaseZap, 70% ethanol, and Gel Slick Solution, was then placed on the slide over the gel embedding solution. Gel embedding solution was allowed to solidify for 90 minutes, after which the coverslip was removed. The sample was then incubated at 37°C in Clearing Solution, comprised of Protease K (New England Biolabs: P8107S) and Clearing Premix (Vizgen: 20300003), for a minimum of 24 hours and up to five days prior to imaging.

On the day of imaging, the slides were washed twice with Sample Preparation Wash Buffer at room temperature and treated with DAPI and PolyT Staining Reagent (Vizgen: 20300021) for 15 minutes on a rocker. The slides were then washed with Formamide Wash Buffer for 15 minutes, followed by a final wash with Sample Prep Wash Buffer. To begin the imaging process, an individual slide was assembled into the MERSCOPE Flow Chamber and inserted into the instrument, along with a MERSCOPE 500 Gene Imaging Cartridge (Vizgen: 20300019). After defining the regions of interest on the slide within the Vizgen MERSCOPE Instrument Software, we started the fully automated instrument run. The MERSCOPE Instrument Software automatically processed the raw images to generate spatial genomics data ready for downstream analysis. Although MERFISH was successful, Slides 3 and 6 underwent unsuccessful Vizgen MERSCOPE protein staining, and these protein staining results were excluded from downstream analyses.

### MERFISH image analysis and cell segmentation:

After image acquisition, the data were initially processed by Vizgen MERSCOPE Instrument Software, before custom cell segmentation was performed with the deep learning algorithm, Cellpose 2.0^50^, using DAPI and PolyT-stained images as training files. First, we uploaded a field of view from one PVH section (Slide 3, bregma level −0.8) as an initial training image. Next, we employed the generalizable ‘cyto2’ model in Cellpose 2.0 with a diameter parameter of 123.73 pixels to initially segment various cell types in the PVH and surrounding regions. Manual annotations were then adjusted by correcting misidentified cells and adding cells missed by the automated ‘cyto2’ model. This process was repeated for 10 fields of view, and the new set of 10 human-processed images were used to optimize the training of our custom Cellpose 2.0 segmentation model. This enhanced model was then utilized to segment cells in all Z planes across 41 coronal sections using the Vizgen Post-processing Tool (VPT). All regions underwent 7-layer segmentation, except for the section corresponding to bregma level −0.7 mm on Slide 2, which underwent segmentation with 6 layers of DAPI and PolyT images due to the loss of the DAPI image from layer 3 during data transfer. Four output files were generated for each coronal section: 1) cellpose2_micron_to_mosaic.parquat (cell boundaries file); 2) cell_by_gene.csv (cell by gene matrix)l; 3) detected_transcripts.csv (cartesian coordinates of each transcript); and 4) cell_metadata.csv (cell morphology characteristics).

### MERFISH sequential gene panel preprocessing:

Due to high-expression levels within the PVH, *Avp*, *Oxt*, and *Sst* expression was assayed with a non-combinatorial sequential gene panel as noted above. Using the VPT sum_signal command on data segmented by Cellpose 2.0, we generated summed fluorescent values for *Avp*, *Oxt*, and *Sst* for each cell in our MERFISH study. We then performed a volume-based normalization of the fluorescent signals using a modified version of previously published methods^127^. Specifically, we first took the High_pass fluorescent values for *Avp*, *Oxt*, and *Sst* for each cell and divided each value by the cell’s volume to yield volume-normalized fluorescence values. Subsequently, we subtracted the respective median volume-normalized fluorescence value for *Avp*, *Oxt*, and *Sst* from all cells and set any negative values to 0. Finally, we divided our median-subtracted, volume-normalized fluorescence value by 1,000 and appended the resulting values for *Avp*, *Oxt*, and *Sst* expression to the cell_by_gene matrix.

### MERFISH data analysis:

VPT output files were loaded as Seurat objects in an R Studio environment (R v 4.4.1) (Seurat v5.0.1.9001) using the Seurat *LoadVizgen()* function. Data from all 41 sections were then merged into one MERFISH Seurat object. Next, we defined the region of interest (ROI) for each section by selecting the rectangular area 200 μm dorsal, 1000 μm ventral, and 700 μm lateral to the top of the third ventricle. The unique IDs for all cells within each ROI detected in *z*-plane three were exported to a .csv file using the Vizgen MERSCOPE Visualizer. The merged MERFISH Seurat object was then subset to retain only cells within our defined ROIs. Subsequently, all cells with less than 15 gene counts were removed, and the remaining cells were analyzed with the Seurat-based pipeline described above, with minor modifications. Notably, *i)* during *FindVariableFeatures()*, clip. range was set to “(−10, 10)”, according to Seurat recommendations for analyzing FISH-based counts, *ii)* no covariates were regressed during scaling of variable features, and *iii)* PCA was conducted with only the combinatorial smFISH features, excluding mCherry. As with sc/snRNA-seq, the merged Seurat MERFISH object was split by ROI (“Slide_ID”) after running PCA, and we subsequently performed a reciprocal principal component analysis (RPCA)-based integration^36^ with Seurat *IntegrateLayers()* to correct for any batch effects. After integration, multiplet clusters driven by inaccurate cell segmentation were removed, and the post-integration steps in our pipeline were repeated until no multiplet clusters were observed. For differential gene expression analysis, we joined layers and ran *FindAllMarkers*() using the non-parametric *Wilcoxon Rank Sum test*. Differentially expressed genes were defined as those > 0.2 average log_2_ fold change and a *Bonferroni-corrected* p-value < 0.01. The post-integration pipeline was run for all levels of subclustering, beginning with all cells, followed by analysis of *Slc17a6*^+^/*Sim1*^+^, *Slc17a6*^+^/*Sim1*^−^, and *Slc32a1*^+^ populations.

### MERFISH Spatial Domain Analysis:

After cell-type clustering with Seurat, we performed a multi-slice spatial domain detection analysis using the R package SpaDo^51^. Due to computational processing limitations, the initial analysis was limited to data from three animals (two male and one female), which had the most extensive rostral-to-caudal coverage of the PVH region and included 25 out of the total 41 tissue slices of the MERFISH analysis. Specifically, we selected slices spanning bregma levels −0.4 mm to −1.2 mm from Slides 3, 4, and 5. Spatial domain analysis was performed by using the *SpatialCellTypeDistribution_multiple*() function to calculate the Spatially Adjacent Cell type Embedding (SPACE) for the MERFISH data. SPACE is calculated via a k-nearest neighbor analysis that identifies a cell’s local niche, which is then integrated with its cell-type annotation derived from the Seurat analysis. Once SPACE was computed, we used the *DistributionDistance*() function to assess similarities between local niches, quantified by Jensen-Shannon divergence (JSD). Subsequently, the *DomainHclust*() function was used with ‘auto_resolution’ set to 1, to derive spatial domains across all included cells and tissue sections. We then imported the calculated spatial domain information into Seurat as metadata to facilitate figure generation. Finally, to allow visualization of spatial domains across all tissue slices, we leveraged the results from this initial analysis to perform reference-based spatial domain annotation of the remaining 16 tissue slices. To accomplish this, we used the *SpatialReference()* and *SpatialQuery()* functions to assign spatial domain annotations to a query dataset based on JSD-distance between the SPACE of each cell in the query dataset and the SPACE centroid for each domain in the reference dataset (**Extended Data Fig. 6a**).

### Stereotaxic injections and optic fiber implantation:

Mice aged 6–10 weeks were deeply anesthetized by intraperitoneal injection of a ketamine/xylazine cocktail (100 mg/kg ketamine; 10 mg/kg xylazine). Next, the surgical area was shaved and sterilized prior to placing the mouse into a stereotactic frame (David Kopf model 940). For spinal cord injections, a midline incision was made above the interscapular region. Vertebrae were visualized by blunt dissection, and T2 was used to identify the injection site location between T2 and T3. The dorsal part of one vertebra was removed with forceps, allowing access to the spinal cord for injection. Injections were made ± 0.4 mm lateral to the midline by lowering a pulled glass pipette containing adeno-associated virus (AAV) or retrograde tracer (Fluoro-Gold or cholera toxin subunit B) into the spinal cord and using an air pressure injection system controlled by a Grass S48 stimulator to control injection speed^128^. Spinal cord injections began at −0.9 mm ventral to the surface of the spinal cord, and AAV/tracer continued to be injected while slowly raising the glass pipette to −0.2 mm. At the completion of each injection, the pipette was left in place for five minutes before removal. This process was then repeated on the contralateral side. To close the incision, the muscle layer was sutured with absorbable sutures (MedVet International: JORG22419), and the skin was sutured with non-absorbable sutures (MedVet International: MV-8661-V). For brain injections, a midline incision was made to expose the skull. At the site of injection, a small hole was drilled, and a pulled glass micropipette containing AAV or retrograde tracer was lowered to the desired injection site depth before infusions commenced using the air pressure injection system described above. Stereotactic coordinates for brain injections were as follows (from bregma): PVH, posterior −0.85, lateral ± 0.2, and ventral −4.9; PB, posterior −5.25, lateral ±1.35, and ventral −3.4; ARC, posterior −1.45, lateral ± 0.3, ventral −6.1. After an injection was completed, the pipette was left in place for five minutes before removal, and this process was repeated for other injection sites. After the injections were completed, the incision was closed using veterinary tissue adhesive (3M Vetbond). For optic fiber implantation, small holes were drilled and 200 μm core fiber optic cannulae with ceramic ferrules (RWD Life Science) were lowered into the PB (posterior, −5.25, lateral ±1.5, and ventral −3.1 from bregma). To secure the cannula, a mixture of dental acrylic and adhesive (dental cement) was then applied to cover the bottom of the ceramic ferrule and the entire exposed area of the skull, anchoring the fiber optic cannulae to the skull. Once the cement had hardened, a non-absorbable suture was placed at the back of the incision to tighten the skin around the cement. After removing the mouse from the stereotaxic frame, the cannula was capped to prevent debris from entering. After surgery, mice were injected with Meloxicam subcutaneously at a dose of 4mg/kg and placed on a 37°C heating pad until recovered.

### AAV and retrograde tracer injections:

For projection-specific sequencing experiments, AAVrg-hSyn-Cre (Addgene: 105553) or HSV-hEf1a-mCherry-IRES-Cre (Mass General Brigham Gene Delivery Technology Core; Dr. Rachael Neve) was injected into the thoracic spinal cord (200 nl/side) or parabrachial region (100 nl/side) of H2B-TRAP mice. Spinal cord retrograde tracing histology was performed by injecting wild-type mice with Fluoro-Gold (FG; Fluorochrome) into the thoracic spinal cord (200 nl/side). For Cre-dependent EGFP-L10a expression in PVH^*Mc4r*^ neurons, *Mc4r*-2a-Cre mice received injections of AAV5-EF1a-FLEX-EGFP-L10a (Addgene: 98747) into the PVH (100 nl/side). These same mice received cholera toxin subunit B (CTB; List Biological Laboratories: 104) injections into the PB (50 nl/side). AAVDJ-hSyn-DIO-EGFP-TeTxLC (ETH Zurich Viral Vector Facility: v322–5) or AAV8-hSyn-DIO-EGFP (control virus; Addgene: 50457) was used for chronic Cre-dependent neuronal silencing experiments via injections into the PVH of *Brs3*-IRES-Cre or wild-type mice (15 nl/side). For CRACM electrophysiology experiments, Cre-dependent AAV8-hSyn-DIO-mCherry (Addgene: 50459) was injected into the PVH (50 nl/side) and Flp-dependent AAV5-EF1a-fDIO-ChR2-eYFP (UNC viral vector core: 172055) was injected into the ARC (200 nl/side) of *Brs3*-IRES-Cre::*Npy*-IRES-Flp mice. *In vivo* optogenetic terminal stimulation experiments were done by injecting Cre-dependent AAV9-EF1a-DIO-ChR2-eYFP (Addgene: 20298) or AAV9-EF1a-DIO-ChR2-mCherry (Addgene: 20297) into the PVH (15 nl/side) of *Brs3*-IRES-Cre mice. Control virus for the optogenetic terminal stimulation experiments was Cre-dependent AAV8-hSyn-DIO-mCherry. Of note, one round of snRNA-seq with 10X Chromium was done by injecting the PVH (50 nl/side) of *Sim1*-Cre mice with AAVDJ-hSyn-H2B-mCherry (Boston Children’s Hospital Viral Core)^119^ and collecting mCherry-positive nuclei^117,118^. Also, the male mouse used for MERFISH Slide 3 was injected with AAVrg-hSyn-mCherry into the spinal cord (200 nl/side), and the male mouse used for MERFISH Slide 6 was injected with AAVrg-hSyn-mCherry into the parabrachial region (50 nl/side). After stereotactic injections, experiments were initiated three weeks post-surgery for all AAVs to allow for suitable expression levels. FG and CTB were injected 3–7 days before sacrifice to enable retrograde transport. All stereotaxic injection sites were validated by post hoc immunofluorescence. All “misses” or “partial” hits, as determined by fluorescent expression in the target cells, were excluded from data analysis.

### RNAscope fluorescent *in situ* hybridization and immunofluorescence:

RNAscope Multiplex Fluorescent Reagent Kit V2 (Advanced Cell Diagnostics: 323100) was used to perform *in situ* hybridization of mRNA in the PVH. For neuroendocrine PVH neuron labeling paired with FISH, adult mice aged 8–12 weeks were injected intraperitoneally with Fluoro-Gold (Fluorochrome; 30 mg/kg) one week prior to lethal injection of ketamine/xylazine (150mg/kg ketamine + 15 mg/kg xylazine) and transcardial perfusion with RNase-free PBS and 10% phosphate-buffered formalin (Fisher: SF100–20). Brains were then extracted and post-fixed in 10% phosphate-buffered formalin overnight, followed by consecutive overnight incubations in 10%, 20%, and 30% RNase-free sucrose solution in PBS. Coronal brain sections were then sliced at 30 μm using a freezing microtome, briefly washed in RNase-free 0.5% Triton X-100 (Sigma Aldrich) in PBS, mounted onto Superfrost^™^ Plus slides, and stored at −80°C until ready for FISH. RNAscope was completed according to the manufacturer’s protocol. First, the slides were removed from the freezer and washed with sterile PBS, followed by a 30-minute incubation at 60°C. The slides were then fixed again with 10% phosphate-buffered formalin for 15 minutes at 4°C, followed by dehydration in 50%, 70%, and 100% ethanol solutions. Hydrogen Peroxide was then added to slides for 10 minutes at room temperature. After washing twice with PBS, a hydrophobic barrier surrounding the tissue sections was drawn on the slide (ImmEdge^™^: H-4000), and the slide was treated with Protease III for 30 minutes. Slides were next hybridized with RNAscope probes targeting mRNA for genes of interest for two hours at 40°C, including *Aox3* (Mm-Aox3: 836451-C1), *Avp* (Mm-Avp: 401391-C3), *Brs3* (Mm-Brs3: 454111-C1 or C3), *Col12a1* (Mm-Col12a1: 312631-C2), *Crh* (Mm-Crh: 316091-C1), *Esr2* (Mm-Esr2: 316121-C3), *Nfix* (Mm-Nfix: 522331-C2), *Npr3* (Mm-Npr3: 502991-C2), *Npsr1* (Mm-Npsr1: 317501-C1), *Oxt* (Mm-Oxt: 493171-C2), *Pla2r1* (Mm-Pla2r1-No-XHs: 854581-C1), *Rxfp3* (Mm-Rxfp3: 439381-C1), *Scgn* (Mm-Scgn: 482721-C2), *Sim2* (Mm-Sim2: 1108911-C1), *Sst* (Mm-Sst: 404631-C1), or *Trh* (Mm-Trh, 436811-C1).After hybridization, slides underwent three amplification steps at 40°C (AMP1-FL and AMP2-FL for 30 minutes each, AMP3-FL for 15 minutes), followed by probe-specific HRP amplification and Opal dye (Akoya Biosciences) incubations at 40°C for visualization. After the Opal dye step, HRP blocker was applied, and this process was repeated until all probes were developed.

After completing RNAscope slides were washed three times with PBS and incubated overnight at 4°C with primary antibody prepared in blocking solution made with PBS, 0.4% Triton X-100, and 3% normal donkey serum. The primary antibodies used include rabbit anti-Fluoro-Gold (1:300; Fluorochrome), goat anti-cholera toxin subunit B (1:300: List Biological Laboratories: 703), and rabbit anti-GFP (1:1,000; Thermo Fisher Scientific: A-11122). The next day, slides were washed five times with PBS, and incubated for two hours at room temperature in the appropriate Alex Fluor-conjugated donkey secondary antibody (1:1,000; Thermo Fisher Scientific) prepared in blocking solution. Finally, slides were washed again three times with PBS before coverslipping with VECTASHIELD mounting media with DAPI (Vector Laboratories: H-1900–10). Slides were imaged at 10X magnification with an Olympus Slideview VS200 slide-scanning microscope or at 20X magnification with a Leica Stellaris 5 confocal microscope.

### Quantification of PVH neuroendocrine neurons:

For each neuroendocrine subtype, 12 images (for *Crh*, *Trh* and *Sst*) and 8 images (for *Avp* and *Oxt*) covering rostral to caudal PVH, were exported using QuPath^129^ from the RNAscope and ip Fluoro-Gold labeling experiments (Fig. 1k–p), which consisted of three channels: Fluoro-Gold, the neuroendocrine hormone of interest, and the novel marker gene for the corresponding neuroendocrine subtype identified by sc/snRNA-seq. Neuroendocrine peptide gene-positive cells were identified using the Cellpose2 model (“cyto2”), with manual adjustments made for any misidentified or missed cells. The selected cell masks were saved and imported into Fiji (ImageJ)^130^, where the multi-point tool further facilitated counting of neurons expressing neuroendocrine marker gene pairs (*Crh-Scgn*, *Trh-Nfix*, *Sst-Col12a1*, *Avp-Pla2r1*, and *Oxt-Rxfp3*) and whether they were labeled by Fluoro-Gold. The percentage of FG-positive neurons for each neuroendocrine marker gene pair was then calculated. The same method is applied to count FG-negative neurons that expressed neuroendocrine peptide genes (FG-negative *Crh*, *Trh*, *Sst*, *Avp* and *Oxt* ) and whether they co-expressed the associated neuroendocrine marker gene identified by sc/snRNA-seq.

### Histological analysis of Cre-reporters and immunofluorescent experiments:

At the conclusion of experiments involving Cre-reporter expression, retrograde tracer injections, and AAV injections, brain/spinal cord histology was performed. For Cre-reporter histology, R26-LSL-EGFP-L10a reporter mice were crossed with *Slc17a6* (VGLUT2)-IRES-Cre, *Slc32a1* (VGAT)-IRES-Cre, and *Sim1*-Cre mice. Adult mice were lethally anesthetized and transcardially perfused as above. Brains were then extracted and postfixed overnight in 10% phosphate-buffered formalin. Brains were then sliced coronally at 40 μm and mounted directly onto glass slides. For experiments requiring immunofluorescence, floating sections were washed in PBS prior to incubation overnight at room temperature in primary antibody solution as described above. All primary antibodies used are described above, except rat anti-mCherry (1:3,000; Thermo Fisher Scientific: M11217). The next day, sections were washed and incubated with Alex Fluor-conjugated donkey secondary antibody as above. Subsequently, tissue was washed, mounted onto slides, and coverslipped with VECTASHIELD mounting media with DAPI. Slides were imaged at 10X magnification with an Olympus Slideview VS200 slide-scanning microscope.

### Body weight measurements after PVH^*Brs3*^ neuron silencing:

To begin bodyweight studies, initial body weights were recorded for littermate *Brs3*-IRES-Cre and wild-type mice, and mice were then divided into the stereotactic surgery groups described above (AAVDJ-hSyn-DIO-EGFP-TeTxLC or AAV8-hSyn-DIO-EGFP). Subsequently, mice remained group-housed for the duration of the experiment. Body weights were recorded during the light cycle between 10:00 AM and 12:00 PM every 7 days for a total of 6 weeks. At the end of the study, mice were transcardially perfused as above for histological analysis of AAV expression in the PVH. Mice without bilateral expression of GFP were removed from the analysis.

### Channelrhodopsin-2 (ChR2)-assisted circuit mapping (CRACM):

*Brs3*-IRES-Cre::*Npy*-IRES-Flp mice underwent stereotactic surgery at 5–7 weeks old as described above, and CRACM experiments were completed at 8–10 weeks-old as described previously^131^. Briefly, mice were anesthetized with isoflurane, decapitated, and brains were rapidly extracted and submerged in ice-cold choline-based cutting solution saturated with carbogen (95% O_2,_ 5% CO_2_). For slice preparation, brains were sliced at 300 μM coronally with a vibrotome (Campden 7000smz-2) and kept in cutting solution at 34°C for 10 min. Next, slices were transferred to artificial cerebrospinal fluid (aCSF) for at least 45 min at room temperature. After recovery, an individual coronal slice containing the PVH region was placed in a recording chamber where it was continuously superfused with aCSF and viewed under a microscope (SliceScope Pro 1000, Scientifica). PVH^*Brs3*^ neurons were fluorescently labeled by Cre-dependent AAV-mCherry, and Flp-dependent AAV-ChR2-eYFP drove ChR2 expression in ARC^*Npy/Agrp*^ neurons. Open-tip resistances for patch pipettes were 3–5 MW and were backfilled with CsCl internal solution. To assess connectivity between ARC^*Npy/Agrp*^ à PVH^*Brs3*^ neurons, whole-cell voltage clamp recordings from PVH^*Brs3*^ neurons were done while photostimulating ChR2-expressing terminals from ARC^*Npy/Agrp*^ neurons. To evoke IPSCs with light, four 470 nm light pulses of 2 ms duration were administered one second apart during the first four seconds of a ten second protocol that was repeated 30 times. Blue light was applied via wide-field exposure through the 40X objective with an LED (Cool LED pE-100). The light output was controlled by a programmable pulse stimulator (Master 8, A.M.P.I.) and pClamp 10.5 software (Axon Instruments). Light-evoked IPSCs were isolated via glutamate receptor antagonism with 1 mM kynurenate, and short latency (≤ 6 ms) responses upon light stimulation were considered to be light-driven.

### Food Intake measurements after PVH^*Brs3*^ neuron à PB optogenetic stimulation:

We assayed dark-cycle food intake while optogenetically stimulating PVH^*Brs3*^ neuron projections to the parabrachial region. *Brs3*-IRES-Cre mice underwent stereotactic surgery for AAV injections and optic fiber implants as described above. Prior to beginning optogenetics studies, mice were allowed to recover for at least three weeks and were acclimated to tethering to patch cords and single housing. On experimental days, patch cords were bilaterally attached to optic fibers over the PB two hours before the onset of dark, and food was removed. Food was returned at the onset of dark and intake was then measured every hour for the first three hours of the dark cycle. Trials consisted of a baseline light-off tests, followed by light-stimulation experimental trials on the following day. Photostimulation was delivered with square wave pulses of 473 nm blue light, delivered at ~8–10 mW of power measured at the fiber tip, with 20 Hz stimulation (10ms pulses; 2 seconds on, 3 seconds off). LabView software and a National Instruments NIDAQ board were used to control our stimulation protocol.

### Statistical analysis

Statistical analyses for sc/snRNA-seq and MERFISH were performed in R, as described above. All other analyses were conducted using GraphPad Prism (v10.3.0), with the specific statistical tests for each experiment indicated in the figure legends. No statistical methods were used to predetermine sample size, and randomization and/or blinding were not applied for sc/snRNA-seq or MERFISH experiments. Randomization was applied for body weight and food intake experiments. For the body weight study, a two-tailed two-way repeated measures ANOVA with virus and time as factors was performed, followed by Tukey’s post hoc multiple comparisons test. For body weight gain measurements, a two-tailed one-way ANOVA followed by Tukey’s post hoc test was used. For the optogenetic feeding behavior assay, a two-tailed two-way repeated measures ANOVA with virus and laser as factors was performed, followed by Sidak’s post hoc multiple comparisons test. All results are presented as mean ± SEM. Statistical significance was defined as P < 0.05, with asterisks indicating significance levels: *P < 0.05, **P < 0.01, and ****P <0.0001.

## Supplementary Material

Supplementary Files

This is a list of supplementary files associated with this preprint. Click to download.

• Lietal.Reportingsummary.pdf

• SupplementaryDataTables.xlsx

• EDFig1.jpg

• EDFig2.jpg

• EDFig3.jpg

• EDFig4.jpg

• EDFig5.jpg

• EDFig6.jpg

• EDFig7.jpg

• EDFig8.jpg

• EDFig9.jpg

• EDFig10.jpg

## Data Availability

This study did not generate any new and unique reagents.

## Data availability

Mouse sc/snRNA-seq data from this study have been deposited into the NCBI Gene Expression Omnibus (GEO) with accession number GSE303256. Allen Brain Cell Atlas mouse sc/snRNA-seq data were downloaded from the following locations: https://allen-brain-cell-atlas.s3.us-west-2.amazonaws.com/index.html#expression_matrices/WMB-10Xv2/20230630/ (10Xv2), and https://allen-brain-cell-atlas.s3.us-west-2.amazonaws.com/index.html#expression_matrices/WMB-10Xv3/20230630/ (10Xv3). Spinal cord-projecting snRNA-seq data not generated in this study were obtained by accessing publicly available datasets within the GEO database: GSE247594^89^ and GSE212409^90^. Human snRNA-seq data were obtained by downloading publicly available datasets, including the Siletti et al.^75^ dataset from the Human Brain Cell Atlas Repository (https://datasets.cellxgene.cziscience.com/5e399d37-23d3-4673-8761-9f443c1fdc14.rds) and the Tadross et al.^76^ dataset from the University of Cambridge Apollo Repository (https://www.repository.cam.ac.uk/items/cad1c61a-e4e5-4443-ad11-92e4f48b3861). Mouse MERFISH data from this study have been deposited in the Iowa Research Online (IRO) repository with record accession number 9984403060302771. All Seurat objects have been deposited to Zenodo at: https://zenodo.org/records/15983704. Any additional information required to reanalyze the data reported in this paper is available from the lead contact upon request.

## Code availability

All custom code has been deposited to Zenodo at: https://zenodo.org/records/15983704. Any additional information required to reanalyze the data reported in this paper is available from the lead contact upon request.

## References

[1] Biag J., Huang Y., Gou L., Hintiryan H., Askarinam A., Hahn J.D., Toga A.W., and Dong H.W. (2012). Cyto- and chemoarchitecture of the hypothalamic paraventricular nucleus in the C57BL/6J male mouse: a study of immunostaining and multiple fluorescent tract tracing. J Comp Neurol 520, 6–33. 10.1002/cne.22698. 21674499 PMC4104804

[2] Swanson L.W., and Kuypers H.G. (1980). The paraventricular nucleus of the hypothalamus: cytoarchitectonic subdivisions and organization of projections to the pituitary, dorsal vagal complex, and spinal cord as demonstrated by retrograde fluorescence double-labeling methods. J Comp Neurol 194, 555–570. 10.1002/cne.901940306. 7451682

[3] Simmons D.M., and Swanson L.W. (2009). Comparison of the spatial distribution of seven types of neuroendocrine neurons in the rat paraventricular nucleus: toward a global 3D model. J Comp Neurol 516, 423–441. 10.1002/cne.22126. 19655400

[4] Sawchenko P.E., and Swanson L.W. (1982). Immunohistochemical identification of neurons in the paraventricular nucleus of the hypothalamus that project to the medulla or to the spinal cord in the rat. J Comp Neurol 205, 260–272. 10.1002/cne.902050306. 6122696

[5] Swanson L.W., Sawchenko P.E., Wiegand S.J., and Price J.L. (1980). Separate neurons in the paraventricular nucleus project to the median eminence and to the medulla or spinal cord. Brain Res 198, 190–195. 10.1016/0006-8993(80)90354-6. 7407584

[6] Swanson L.W., and Sawchenko P.E. (1980). Paraventricular nucleus: a site for the integration of neuroendocrine and autonomic mechanisms. Neuroendocrinology 31, 410–417. 10.1159/000123111. 6109264

[7] Armstrong W.E., Warach S., Hatton G.I., and McNeill T.H. (1980). Subnuclei in the rat hypothalamic paraventricular nucleus: a cytoarchitectural, horseradish peroxidase and immunocytochemical analysis. Neuroscience 5, 1931–1958. 10.1016/0306-4522(80)90040-8. 7432630

[8] Swanson L.W. (1977). Immunohistochemical evidence for a neurophysin-containing autonomic pathway arising in the paraventricular nucleus of the hypothalamus. Brain Res 128, 346–353. 10.1016/0006-8993(77)91000-9. 301423

[9] Saper C.B., Loewy A.D., Swanson L.W., and Cowan W.M. (1976). Direct hypothalamo-autonomic connections. Brain Res 117, 305–312. 10.1016/0006-8993(76)90738-1. 62600

[10] Geerling J.C., Shin J.W., Chimenti P.C., and Loewy A.D. (2010). Paraventricular hypothalamic nucleus: axonal projections to the brainstem. J Comp Neurol 518, 1460–1499. 10.1002/cne.22283. 20187136 PMC2868510

[11] Krashes M.J., Lowell B.B., and Garfield A.S. (2016). Melanocortin-4 receptor–regulated energy homeostasis. Nature Neuroscience 19, 206–219. 10.1038/nn.4202. 26814590 PMC5244821

[12] Garfield A.S., Li C., Madara J.C., Shah B.P., Webber E., Steger J.S., Campbell J.N., Gavrilova O., Lee C.E., Olson D.P., (2015). A neural basis for melanocortin-4 receptor-regulated appetite. Nat Neurosci 18, 863–871. 10.1038/nn.4011. 25915476 PMC4446192

[13] Shah B.P., Vong L., Olson D.P., Koda S., Krashes M.J., Ye C., Yang Z., Fuller P.M., Elmquist J.K., and Lowell B.B. (2014). MC4R-expressing glutamatergic neurons in the paraventricular hypothalamus regulate feeding and are synaptically connected to the parabrachial nucleus. Proc Natl Acad Sci U S A 111, 13193–13198. 10.1073/pnas.1407843111. 25157144 PMC4246954

[14] Balthasar N., Dalgaard L.T., Lee C.E., Yu J., Funahashi H., Williams T., Ferreira M., Tang V., McGovern R.A., Kenny C.D., (2005). Divergence of melanocortin pathways in the control of food intake and energy expenditure. Cell 123, 493–505. 10.1016/j.cell.2005.08.035. 16269339

[15] Li C., Navarrete J., Liang-Guallpa J., Lu C., Funderburk S.C., Chang R.B., Liberles S.D., Olson D.P., and Krashes M.J. (2019). Defined Paraventricular Hypothalamic Populations Exhibit Differential Responses to Food Contingent on Caloric State. Cell Metab 29, 681–694 e685. 10.1016/j.cmet.2018.10.016. 30472090 PMC6402975

[16] Gonzalez I.E., Ramirez-Matias J., Lu C., Pan W., Zhu A., Myers M.G., and Olson D.P. (2021). Paraventricular Calcitonin Receptor-Expressing Neurons Modulate Energy Homeostasis in Male Mice. Endocrinology 162. 10.1210/endocr/bqab072. PMC8139622 33834205

[17] Sutton A.K., Gonzalez I.E., Sadagurski M., Rajala M., Lu C., Allison M.B., Adams J.M., Myers M.G., White M.F., and Olson D.P. (2020). Paraventricular, subparaventricular and periventricular hypothalamic IRS4-expressing neurons are required for normal energy balance. Sci Rep 10, 5546. 10.1038/s41598-020-62468-z. 32218485 PMC7099088

[18] An J.J., Kinney C.E., Tan J.W., Liao G.Y., Kremer E.J., and Xu B. (2020). TrkB-expressing paraventricular hypothalamic neurons suppress appetite through multiple neurocircuits. Nat Commun 11, 1729. 10.1038/s41467-020-15537-w. 32265438 PMC7138837

[19] Sutton A.K., Pei H., Burnett K.H., Myers M.G. Jr., Rhodes C.J., and Olson D.P. (2014). Control of food intake and energy expenditure by Nos1 neurons of the paraventricular hypothalamus. J Neurosci 34, 15306–15318. 10.1523/JNEUROSCI.0226-14.2014. 25392498 PMC4228133

[20] Pei H., Sutton A.K., Burnett K.H., Fuller P.M., and Olson D.P. (2014). AVP neurons in the paraventricular nucleus of the hypothalamus regulate feeding. Mol Metab 3, 209–215. 10.1016/j.molmet.2013.12.006. 24634830 PMC3953699

[21] Li M.M., Madara J.C., Steger J.S., Krashes M.J., Balthasar N., Campbell J.N., Resch J.M., Conley N.J., Garfield A.S., and Lowell B.B. (2019). The Paraventricular Hypothalamus Regulates Satiety and Prevents Obesity via Two Genetically Distinct Circuits. Neuron 102, 653–667 e656. 10.1016/j.neuron.2019.02.028. 30879785 PMC6508999

[22] Krashes M.J., Shah B.P., Madara J.C., Olson D.P., Strochlic D.E., Garfield A.S., Vong L., Pei H., Watabe-Uchida M., Uchida N., (2014). An excitatory paraventricular nucleus to AgRP neuron circuit that drives hunger. Nature 507, 238–242. 10.1038/nature12956. 24487620 PMC3955843

[23] An J.J., Liao G.Y., Kinney C.E., Sahibzada N., and Xu B. (2015). Discrete BDNF Neurons in the Paraventricular Hypothalamus Control Feeding and Energy Expenditure. Cell Metab 22, 175–188. 10.1016/j.cmet.2015.05.008. 26073495 PMC4497865

[24] Wang P., Loh K.H., Wu M., Morgan D.A., Schneeberger M., Yu X., Chi J., Kosse C., Kim D., Rahmouni K., (2020). A leptin–BDNF pathway regulating sympathetic innervation of adipose tissue. Nature 583, 839–844. 10.1038/s41586-020-2527-y. 32699414

[25] Berkhout J.B., Poormoghadam D., Yi C., Kalsbeek A., Meijer O.C., and Mahfouz A. (2024). An integrated single-cell RNA-seq atlas of the mouse hypothalamic paraventricular nucleus links transcriptomic and functional types. J Neuroendocrinol, e13367. 10.1111/jne.13367. 38281730

[26] Xu S., Yang H., Menon V., Lemire A.L., Wang L., Henry F.E., Turaga S.C., and Sternson S.M. (2020). Behavioral state coding by molecularly defined paraventricular hypothalamic cell type ensembles. Science 370. 10.1126/science.abb2494. PMC11938375 33060330

[27] Romanov R.A., Alpar A., Zhang M.D., Zeisel A., Calas A., Landry M., Fuszard M., Shirran S.L., Schnell R., Dobolyi A., (2015). A secretagogin locus of the mammalian hypothalamus controls stress hormone release. EMBO J 34, 36–54. 10.15252/embj.201488977. 25430741 PMC4291479

[28] Zhang M., Pan X., Jung W., Halpern A.R., Eichhorn S.W., Lei Z., Cohen L., Smith K.A., Tasic B., Yao Z., (2023). Molecularly defined and spatially resolved cell atlas of the whole mouse brain. Nature 624, 343–354. 10.1038/s41586-023-06808-9. 38092912 PMC10719103

[29] Yao Z., van Velthoven C.T.J., Kunst M., Zhang M., McMillen D., Lee C., Jung W., Goldy J., Abdelhak A., Aitken M., (2023). A high-resolution transcriptomic and spatial atlas of cell types in the whole mouse brain. Nature 624, 317–332. 10.1038/s41586-023-06812-z. 38092916 PMC10719114

[30] Shi H., He Y., Zhou Y., Huang J., Maher K., Wang B., Tang Z., Luo S., Tan P., Wu M., (2023). Spatial atlas of the mouse central nervous system at molecular resolution. Nature. 10.1038/s41586-023-06569-5. PMC10709140 37758947

[31] Langlieb J., Sachdev N.S., Balderrama K.S., Nadaf N.M., Raj M., Murray E., Webber J.T., Vanderburg C., Gazestani V., Tward D., (2023). The molecular cytoarchitecture of the adult mouse brain. Nature 624, 333–342. 10.1038/s41586-023-06818-7. 38092915 PMC10719111

[32] Steuernagel L., Lam B.Y.H., Klemm P., Dowsett G.K.C., Bauder C.A., Tadross J.A., Hitschfeld T.S., del Rio Martin A., Chen W., de Solis A.J., (2022). HypoMap—a unified single-cell gene expression atlas of the murine hypothalamus. Nature Metabolism 4, 1402–1419. 10.1038/s42255-022-00657-y. PMC9584816 36266547

[33] Macosko E.Z., Basu A., Satija R., Nemesh J., Shekhar K., Goldman M., Tirosh I., Bialas A.R., Kamitaki N., Martersteck E.M., (2015). Highly Parallel Genome-wide Expression Profiling of Individual Cells Using Nanoliter Droplets. Cell 161, 1202 – 1214. 10.1016/j.cell.2015.05.002. 26000488 PMC4481139

[34] Habib N., Avraham-Davidi I., Basu A., Burks T., Shekhar K., Hofree M., Choudhury S.R., Aguet F., Gelfand E., Ardlie K., (2017). Massively parallel single-nucleus RNA-seq with DroNc-seq. Nature Methods 14, 955 – 958. 10.1038/nmeth.4407. 28846088 PMC5623139

[35] Hao Y., Stuart T., Kowalski M.H., Choudhary S., Hoffman P., Hartman A., Srivastava A., Molla G., Madad S., Fernandez-Granda C., and Satija R. (2024). Dictionary learning for integrative, multimodal and scalable single-cell analysis. Nat Biotechnol 42, 293–304. 10.1038/s41587-023-01767-y. 37231261 PMC10928517

[36] Stuart T., Butler A., Hoffman P., Hafemeister C., Papalexi E., Mauck W.M. 3rd, Hao Y., Stoeckius M., Smibert P., and Satija R. (2019). Comprehensive Integration of Single-Cell Data. Cell 177, 1888–1902 e1821. 10.1016/j.cell.2019.05.031. 31178118 PMC6687398

[37] Cullinan W.E., Ziegler D.R., and Herman J.P. (2008). Functional role of local GABAergic influences on the HPA axis. Brain Struct Funct 213, 63–72. 10.1007/s00429-008-0192-2. 18696110

[38] Roland B.L., and Sawchenko P.E. (1993). Local origins of some GABAergic projections to the paraventricular and supraoptic nuclei of the hypothalamus in the rat. J Comp Neurol 332, 123–143. 10.1002/cne.903320109. 7685780

[39] Michaud J.L., Rosenquist T., May N.R., and Fan C.M. (1998). Development of neuroendocrine lineages requires the bHLH-PAS transcription factor SIM1. Genes Dev 12, 3264–3275. 10.1101/gad.12.20.3264. 9784500 PMC317216

[40] Lein E.S., Hawrylycz M.J., Ao N., Ayres M., Bensinger A., Bernard A., Boe A.F., Boguski M.S., Brockway K.S., Byrnes E.J., (2007). Genome-wide atlas of gene expression in the adult mouse brain. Nature 445, 168–176. 10.1038/nature05453. 17151600

[41] Zupancic M., Tretiakov E., Mate Z., Erdelyi F., Szabo G., Clotman F., Hokfelt T., Harkany T., and Keimpema E. (2023). Brain-wide mapping of efferent projections of glutamatergic (Onecut3(+) ) neurons in the lateral mouse hypothalamus. Acta Physiol (Oxf) 238, e13973. 10.1111/apha.13973. 37029761 PMC10909463

[42] Asbreuk C.H., van Schaick H.S., Cox J.J., Smidt M.P., and Burbach J.P. (2002). Survey for paired-like homeodomain gene expression in the hypothalamus: restricted expression patterns of Rx, Alx4 and goosecoid. Neuroscience 114, 883–889. 10.1016/s0306-4522(02)00325-1. 12379244

[43] Merchenthaler I. (1991). Neurons with access to the general circulation in the central nervous system of the rat: a retrograde tracing study with fluoro-gold. Neuroscience 44, 655–662. 10.1016/0306-4522(91)90085-3. 1721686

[44] Ambalavanar R., and Morris R. (1989). Fluoro-Gold injected either subcutaneously or intravascularly results in extensive retrograde labelling of CNS neurones having axons terminating outside the blood-brain barrier. Brain Res 505, 171–175. 10.1016/0006-8993(89)90133-9. 2611674

[45] Minami S., Kamegai J., Sugihara H., Suzuki N., and Wakabayashi I. (1998). Growth hormone inhibits its own secretion by acting on the hypothalamus through its receptors on neuropeptide Y neurons in the arcuate nucleus and somatostatin neurons in the periventricular nucleus. Endocr J 45 Suppl, S19–26. 10.1507/endocrj.45.suppl_s19. 9790225

[46] Nardone S., De Luca R., Zito A., Klymko N., Nicoloutsopoulos D., Amsalem O., Brannigan C., Resch J.M., Jacobs C.L., Pant D., (2024). A spatially-resolved transcriptional atlas of the murine dorsal pons at single-cell resolution. Nat Commun 15, 1966. 10.1038/s41467-024-45907-7. 38438345 PMC10912765

[47] Chen K.H., Boettiger A.N., Moffitt J.R., Wang S., and Zhuang X. (2015). RNA imaging. Spatially resolved, highly multiplexed RNA profiling in single cells. Science 348, aaa6090. 10.1126/science.aaa6090. 25858977 PMC4662681

[48] Hartman A., and Satija R. (2024). Comparative analysis of multiplexed in situ gene expression profiling technologies. Elife. 10.7554/elife.96949.1.

[49] Paxinos G., and Franklin K.B.J. (2019). Paxinos and Franklin’s The mouse brain in stereotaxic coordinates, Fifth edition. Edition (Academic Press, an imprint of Elsevier).

[50] Pachitariu M., and Stringer C. (2022). Cellpose 2.0: how to train your own model. Nat Methods 19, 1634–1641. 10.1038/s41592-022-01663-4. 36344832 PMC9718665

[51] Duan B., Chen S., Cheng X., and Liu Q. (2024). Multi-slice spatial transcriptome domain analysis with SpaDo. Genome Biol 25, 73. 10.1186/s13059-024-03213-x. 38504325 PMC10949687

[52] Goshu E., Jin H., Lovejoy J., Marion J.F., Michaud J.L., and Fan C.M. (2004). Sim2 contributes to neuroendocrine hormone gene expression in the anterior hypothalamus. Mol Endocrinol 18, 1251–1262. 10.1210/me.2003-0372. 14988428

[53] Grzelka K., Wilhelms H., Dodt S., Dreisow M.L., Madara J.C., Walker S.J., Wu C., Wang D., Lowell B.B., and Fenselau H. (2023). A synaptic amplifier of hunger for regaining body weight in the hypothalamus. Cell Metab 35, 770–785 e775. 10.1016/j.cmet.2023.03.002. 36965483 PMC10160008

[54] Wang C.S., Kavalali E.T., and Monteggia L.M. (2022). BDNF signaling in context: From synaptic regulation to psychiatric disorders. Cell 185, 62–76. 10.1016/j.cell.2021.12.003. 34963057 PMC8741740

[55] Tao W., Diaz-Alonso J., Sheng N., and Nicoll R.A. (2018). Postsynaptic delta1 glutamate receptor assembles and maintains hippocampal synapses via Cbln2 and neurexin. Proc Natl Acad Sci U S A 115, E5373–E5381. 10.1073/pnas.1802737115. 29784783 PMC6003362

[56] Seigneur E., Polepalli J.S., and Sudhof T.C. (2018). Cbln2 and Cbln4 are expressed in distinct medial habenula-interpeduncular projections and contribute to different behavioral outputs. Proc Natl Acad Sci U S A 115, E10235–E10244. 10.1073/pnas.1811086115. 30287486 PMC6205418

[57] Liu T., Kong D., Shah B.P., Ye C., Koda S., Saunders A., Ding J.B., Yang Z., Sabatini B.L., and Lowell B.B. (2012). Fasting activation of AgRP neurons requires NMDA receptors and involves spinogenesis and increased excitatory tone. Neuron 73, 511–522. 10.1016/j.neuron.2011.11.027. 22325203 PMC3278709

[58] Walker S.J., Lowenstein E.D., Douglass A.M., Madara J.C., Resch J.M., Tao J., and Lowell B.B. (2025). A hypothalamic circuit for anticipating future changes in energy balance. bioRxiv, 2025.2009.2027.678865. 10.1101/2025.09.27.678865. PMC13353161 42235510

[59] Lowell B.B. (2019). New Neuroscience of Homeostasis and Drives for Food, Water, and Salt. N Engl J Med 380, 459–471. 10.1056/NEJMra1812053. 30699320

[60] Nguyen A.D., Mitchell N.F., Lin S., Macia L., Yulyaningsih E., Baldock P.A., Enriquez R.F., Zhang L., Shi Y.C., Zolotukhin S., (2012). Y1 and Y5 receptors are both required for the regulation of food intake and energy homeostasis in mice. PLoS One 7, e40191. 10.1371/journal.pone.0040191. 22768253 PMC3387009

[61] Isgor C., Cecchi M., Kabbaj M., Akil H., and Watson S.J. (2003). Estrogen receptor beta in the paraventricular nucleus of hypothalamus regulates the neuroendocrine response to stress and is regulated by corticosterone. Neuroscience 121, 837–845. 10.1016/s0306-4522(03)00561-x. 14580933

[62] Gingerich S., and Krukoff T.L. (2006). Estrogen in the paraventricular nucleus attenuates L-glutamate-induced increases in mean arterial pressure through estrogen receptor beta and NO. Hypertension 48, 1130–1136. 10.1161/01.HYP.0000248754.67128.ff. 17075034

[63] Milner T.A., Contoreggi N.H., Yu F., Johnson M.A., Wang G., Woods C., Mazid S., Van Kempen T.A., Waters E.M., McEwen B.S., (2021). Estrogen Receptor beta Contributes to Both Hypertension and Hypothalamic Plasticity in a Mouse Model of Peri-Menopause. J Neurosci 41, 5190–5205. 10.1523/JNEUROSCI.0164-21.2021. 33941651 PMC8211546

[64] Sommer G., Rodriguez Lopez C., Hirschkorn A., Calimano G., Marques-Lopes J., Milner T.A., and Glass M.J. (2024). Estrogen Receptor Beta Agonist Influences Presynaptic NMDA Receptor Distribution in the Paraventricular Hypothalamic Nucleus Following Hypertension in a Mouse Model of Perimenopause. Biology (Basel) 13. 10.3390/biology13100819. PMC11505520 39452127

[65] Zheng H., Patel T.A., Liu X., and Patel K.P. (2023). C-type natriuretic peptide (CNP) in the paraventricular nucleus-mediated renal sympatho-inhibition. Front Physiol 14, 1162699. 10.3389/fphys.2023.1162699. 37082246 PMC10110992

[66] Xiao C., and Reitman M.L. (2016). Bombesin-Like Receptor 3: Physiology of a Functional Orphan. Trends Endocrinol Metab 27, 603–605. 10.1016/j.tem.2016.03.003. 27055378 PMC4992652

[67] Pinol R.A., Zahler S.H., Li C., Saha A., Tan B.K., Skop V., Gavrilova O., Xiao C., Krashes M.J., and Reitman M.L. (2018). Brs3 neurons in the mouse dorsomedial hypothalamus regulate body temperature, energy expenditure, and heart rate, but not food intake. Nat Neurosci 21, 1530–1540. 10.1038/s41593-018-0249-3. 30349101 PMC6203600

[68] Maruyama M., Hotta N., Nio Y., Hamagami K., Nagi T., Funata M., Sakamoto J., Nakakariya M., Amano N., Nishida M., (2018). Bombesin receptor subtype-3-expressing neurons regulate energy homeostasis through a novel neuronal pathway in the hypothalamus. Brain Behav 8, e00881. 10.1002/brb3.881. 29568682 PMC5853643

[69] Ladenheim E.E., Behles R.R., Bi S., and Moran T.H. (2009). Gastrin-releasing peptide messenger ribonucleic acid expression in the hypothalamic paraventricular nucleus is altered by melanocortin receptor stimulation and food deprivation. Endocrinology 150, 672–678. 10.1210/en.2008-0559. 18818295 PMC2646528

[70] Autry A.E., Wu Z., Kapoor V., Kohl J., Bambah-Mukku D., Rubinstein N.D., Marin-Rodriguez B., Carta I., Sedwick V., Tang M., and Dulac C. (2021). Urocortin-3 neurons in the mouse perifornical area promote infant-directed neglect and aggression. Elife 10. 10.7554/eLife.64680. PMC8452308 34423776

[71] van-Hover C., and Li C. (2015). Stress-activated afferent inputs into the anterior parvicellular part of the paraventricular nucleus of the hypothalamus: Insights into urocortin 3 neuron activation. Brain Res 1611, 29–43. 10.1016/j.brainres.2015.03.009. 25779038 PMC4441854

[72] Mihaly E., Fekete C., Tatro J.B., Liposits Z., Stopa E.G., and Lechan R.M. (2000). Hypophysiotropic thyrotropin-releasing hormone-synthesizing neurons in the human hypothalamus are innervated by neuropeptide Y, agouti-related protein, and alpha-melanocyte-stimulating hormone. J Clin Endocrinol Metab 85, 2596–2603. 10.1210/jcem.85.7.6662. 10902813

[73] Gai W.P., Geffen L.B., and Blessing W.W. (1990). Galanin immunoreactive neurons in the human hypothalamus: colocalization with vasopressin-containing neurons. J Comp Neurol 298, 265–280. 10.1002/cne.902980302. 1698834

[74] Krolewski D.M., Medina A., Kerman I.A., Bernard R., Burke S., Thompson R.C., Bunney W.E. Jr., Schatzberg A.F., Myers R.M., Akil H., (2010). Expression patterns of corticotropin-releasing factor, arginine vasopressin, histidine decarboxylase, melanin-concentrating hormone, and orexin genes in the human hypothalamus. J Comp Neurol 518, 4591–4611. 10.1002/cne.22480. 20886624 PMC2965642

[75] Siletti K., Hodge R., Mossi Albiach A., Lee K.W., Ding S.L., Hu L., Lonnerberg P., Bakken T., Casper T., Clark M., (2023). Transcriptomic diversity of cell types across the adult human brain. Science 382, eadd7046. 10.1126/science.add7046. 37824663

[76] Tadross J.A., Steuernagel L., Dowsett G.K.C., Kentistou K.A., Lundh S., Porniece M., Klemm P., Rainbow K., Hvid H., Kania K., (2025). A comprehensive spatio-cellular map of the human hypothalamus. Nature. 10.1038/s41586-024-08504-8. PMC11922758 39910307

[77] Herman J.P., Tasker J.G., Ziegler D.R., and Cullinan W.E. (2002). Local circuit regulation of paraventricular nucleus stress integration: glutamate-GABA connections. Pharmacol Biochem Behav 71, 457–468. 10.1016/s0091-3057(01)00681-5. 11830180

[78] Watts A.G., and Swanson L.W. (1987). Efferent projections of the suprachiasmatic nucleus: II. Studies using retrograde transport of fluorescent dyes and simultaneous peptide immunohistochemistry in the rat. J Comp Neurol 258, 230–252. 10.1002/cne.902580205. 2438309

[79] Stocker S.D., Cunningham J.T., and Toney G.M. (2004). Water deprivation increases Fos immunoreactivity in PVN autonomic neurons with projections to the spinal cord and rostral ventrolateral medulla. Am J Physiol Regul Integr Comp Physiol 287, R1172–1183. 10.1152/ajpregu.00394.2004. 15271657

[80] Pyner S., and Coote J.H. (2000). Identification of branching paraventricular neurons of the hypothalamus that project to the rostroventrolateral medulla and spinal cord. Neuroscience 100, 549–556. 10.1016/s0306-4522(00)00283-9. 11098118

[81] Toth Z.E., Gallatz K., Fodor M., and Palkovits M. (1999). Decussations of the descending paraventricular pathways to the brainstem and spinal cord autonomic centers. J Comp Neurol 414, 255–266. 10516595

[82] Shafton A.D., Ryan A., and Badoer E. (1998). Neurons in the hypothalamic paraventricular nucleus send collaterals to the spinal cord and to the rostral ventrolateral medulla in the rat. Brain Res 801, 239–243. 10.1016/s0006-8993(98)00587-3. 9729407

[83] Zheng J.Q., Seki M., Hayakawa T., Ito H., and Zyo K. (1995). Descending projections from the paraventricular hypothalamic nucleus to the spinal cord: anterograde tracing study in the rat. Okajimas Folia Anat Jpn 72, 119–135. 10.2535/ofaj1936.72.2-3_119. 8559555

[84] Luiten P.G., ter Horst G.J., Karst H., and Steffens A.B. (1985). The course of paraventricular hypothalamic efferents to autonomic structures in medulla and spinal cord. Brain Res 329, 374–378. 10.1016/0006-8993(85)90554-2. 3978460

[85] Zhang Z., Su J., Tang J., Chung L., Page J.C., Winter C.C., Liu Y., Kegeles E., Conti S., Zhang Y., (2024). Spinal projecting neurons in rostral ventromedial medulla co-regulate motor and sympathetic tone. Cell. 10.1016/j.cell.2024.04.022. PMC11193620 38733990

[86] Nunn N., Womack M., Dart C., and Barrett-Jolley R. (2011). Function and pharmacology of spinally-projecting sympathetic pre-autonomic neurones in the paraventricular nucleus of the hypothalamus. Curr Neuropharmacol 9, 262–277. 10.2174/157015911795596531. 22131936 PMC3131718

[87] Carrasco M., Portillo F., Larsen P.J., and Vallo J.J. (2001). Insulin and glucose administration stimulates Fos expression in neurones of the paraventricular nucleus that project to autonomic preganglionic structures. J Neuroendocrinol 13, 339–346. 10.1046/j.1365-2826.2001.00631.x. 11264721

[88] Badoer E., McKinley M.J., Oldfield B.J., and McAllen R.M. (1993). A comparison of hypotensive and non-hypotensive hemorrhage on Fos expression in spinally projecting neurons of the paraventricular nucleus and rostral ventrolateral medulla. Brain Res 610, 216–223. 10.1016/0006-8993(93)91403-f. 8319084

[89] Winter C.C., Jacobi A., Su J., Chung L., van Velthoven C.T.J., Yao Z., Lee C., Zhang Z., Yu S., Gao K., (2023). A transcriptomic taxonomy of mouse brain-wide spinal projecting neurons. Nature 624, 403–414. 10.1038/s41586-023-06817-8. 38092914 PMC10719099

[90] Beine Z., Wang Z., Tsoulfas P., and Blackmore M.G. (2022). Single nuclei analyses reveal transcriptional profiles and marker genes for diverse supraspinal populations. J Neurosci. 10.1523/JNEUROSCI.1197-22.2022. PMC9698772 36202615

[91] Roh H.C., Tsai L.T.Y., Lyubetskaya A., Tenen D., Kumari M., and Rosen E.D. (2017). Simultaneous Transcriptional and Epigenomic Profiling from Specific Cell Types within Heterogeneous Tissues In Vivo. Cell Reports 18, 1048–1061. 10.1016/j.celrep.2016.12.087. 28122230 PMC5291126

[92] Kishi T., Aschkenasi C.J., Choi B.J., Lopez M.E., Lee C.E., Liu H., Hollenberg A.N., Friedman J.M., and Elmquist J.K. (2005). Neuropeptide Y Y1 receptor mRNA in rodent brain: distribution and colocalization with melanocortin-4 receptor. J Comp Neurol 482, 217–243. 10.1002/cne.20432. 15690487

[93] Qi Y., Lee N.J., Ip C.K., Enriquez R., Tasan R., Zhang L., and Herzog H. (2022). NPY derived from AGRP neurons controls feeding via Y1 and energy expenditure and food foraging behaviour via Y2 signalling. Mol Metab 59, 101455. 10.1016/j.molmet.2022.101455. 35167990 PMC8886056

[94] Atasoy D., Betley J.N., Su H.H., and Sternson S.M. (2012). Deconstruction of a neural circuit for hunger. Nature 488, 172–177. 10.1038/nature11270. 22801496 PMC3416931

[95] Vella K.R., Ramadoss P., Lam F.S., Harris J.C., Ye F.D., Same P.D., O’Neill N.F., Maratos-Flier E., and Hollenberg A.N. (2011). NPY and MC4R signaling regulate thyroid hormone levels during fasting through both central and peripheral pathways. Cell Metab 14, 780–790. 10.1016/j.cmet.2011.10.009. 22100407 PMC3261758

[96] Xiao C., Liu N., Province H., Piñol R.A., Gavrilova O., and Reitman M.L. (2020). BRS3 in both MC4R-and SIM1-expressing neurons regulates energy homeostasis in mice. Molecular Metabolism 36, 100969. 10.1016/j.molmet.2020.02.012. 32229422 PMC7113433

[97] Liu J., Conde K., Zhang P., Lilascharoen V., Xu Z., Lim B.K., Seeley R.J., Zhu J.J., Scott M.M., and Pang Z.P. (2017). Enhanced AMPA Receptor Trafficking Mediates the Anorexigenic Effect of Endogenous Glucagon-like Peptide-1 in the Paraventricular Hypothalamus. Neuron 96, 897–909 e895. 10.1016/j.neuron.2017.09.042. 29056294 PMC5729931

[98] Geerling J.C., and Loewy A.D. (2006). Aldosterone-sensitive neurons in the nucleus of the solitary tract: efferent projections. J Comp Neurol 497, 223–250. 10.1002/cne.20993. 16705681

[99] Nectow A.R., Moya M.V., Ekstrand M.I., Mousa A., McGuire K.L., Sferrazza C.E., Field B.C., Rabinowitz G.S., Sawicka K., Liang Y., (2017). Rapid Molecular Profiling of Defined Cell Types Using Viral TRAP. Cell Rep 19, 655–667. 10.1016/j.celrep.2017.03.048. 28423326 PMC5476221

[100] Mogul A.S., Hadley C.K., Province H.S., Pauli J., Gavrilova O., Xiao C., Palmiter R.D., Pinol R.A., and Reitman M.L. (2021). Cre Recombinase Driver Mice Reveal Lineage-Dependent and -Independent Expression of Brs3 in the Mouse Brain. Eneuro 8, ENEURO.0252–0221.2021. 10.1523/ENEURO.0252-21.2021. PMC8371926 34326065

[101] Daigle T.L., Madisen L., Hage T.A., Valley M.T., Knoblich U., Larsen R.S., Takeno M.M., Huang L., Gu H., Larsen R., (2018). A Suite of Transgenic Driver and Reporter Mouse Lines with Enhanced Brain-Cell-Type Targeting and Functionality. Cell 174, 465–480 e422. 10.1016/j.cell.2018.06.035. 30007418 PMC6086366

[102] Hahn T.M., Breininger J.F., Baskin D.G., and Schwartz M.W. (1998). Coexpression of Agrp and NPY in fasting-activated hypothalamic neurons. Nat Neurosci 1, 271–272. 10.1038/1082. 10195157

[103] Iremonger K.J., and Power E.M. (2025). The paraventricular nucleus of the hypothalamus: a key node in the control of behavioural states. J Physiol. 10.1113/JP288366. PMC12013795 40119815

[104] Menon R., and Neumann I.D. (2023). Detection, processing and reinforcement of social cues: regulation by the oxytocin system. Nat Rev Neurosci 24, 761–777. 10.1038/s41583-023-00759-w. 37891399

[105] Rasiah N.P., Loewen S.P., and Bains J.S. (2023). Windows into stress: a glimpse at emerging roles for CRH(PVN) neurons. Physiol Rev 103, 1667–1691. 10.1152/physrev.00056.2021. 36395349

[106] Zhang B., Qiu L., Xiao W., Ni H., Chen L., Wang F., Mai W., Wu J., Bao A., Hu H., (2021). Reconstruction of the Hypothalamo-Neurohypophysial System and Functional Dissection of Magnocellular Oxytocin Neurons in the Brain. Neuron 109, 331–346 e337. 10.1016/j.neuron.2020.10.032. 33212012

[107] Li H., Jiang T., An S., Xu M., Gou L., Ren B., Shi X., Wang X., Yan J., Yuan J., (2024). Single-neuron projectomes of mouse paraventricular hypothalamic nucleus oxytocin neurons reveal mutually exclusive projection patterns. Neuron. 10.1016/j.neuron.2023.12.022. 38290516

[108] Fuzesi T., Daviu N., Wamsteeker Cusulin J.I., Bonin R.P., and Bains J.S. (2016). Hypothalamic CRH neurons orchestrate complex behaviours after stress. Nat Commun 7, 11937. 10.1038/ncomms11937. 27306314 PMC4912635

[109] Rho J.H., and Swanson L.W. (1987). Neuroendocrine CRF motoneurons: intrahypothalamic axon terminals shown with a new retrograde-Lucifer-immuno method. Brain Res 436, 143–147. 10.1016/0006-8993(87)91566-6. 2446717

[110] Kc P., Haxhiu M.A., Tolentino-Silva F.P., Wu M., Trouth C.O., and Mack S.O. (2002). Paraventricular vasopressin-containing neurons project to brain stem and spinal cord respiratory-related sites. Respir Physiol Neurobiol 133, 75–88. 10.1016/s1569-9048(02)00131-3. 12385733

[111] Oti T., Satoh K., Uta D., Nagafuchi J., Tateishi S., Ueda R., Takanami K., Young L.J., Galione A., Morris J.F., (2021). Oxytocin Influences Male Sexual Activity via Non-synaptic Axonal Release in the Spinal Cord. Curr Biol 31, 103–114 e105. 10.1016/j.cub.2020.09.089. 33125871 PMC7855431

[112] Papazoglou I., Lee J.H., Cui Z., Li C., Fulgenzi G., Bahn Y.J., Staniszewska-Goraczniak H.M., Pinol R.A., Hogue I.B., Enquist L.W., (2022). A distinct hypothalamus-to-beta cell circuit modulates insulin secretion. Cell Metab 34, 285–298 e287. 10.1016/j.cmet.2021.12.020. 35108515 PMC8935365

[113] Chen S., and Aston-Jones G. (1995). Evidence that cholera toxin B subunit (CTb) can be avidly taken up and transported by fibers of passage. Brain Res 674, 107–111. 10.1016/0006-8993(95)00020-q. 7773677

[114] Vong L., Ye C., Yang Z., Choi B., Chua S. Jr., and Lowell B.B. (2011). Leptin action on GABAergic neurons prevents obesity and reduces inhibitory tone to POMC neurons. Neuron 71, 142–154. 10.1016/j.neuron.2011.05.028. 21745644 PMC3134797

[115] Pauli J.L., Chen J.Y., Basiri M.L., Park S., Carter M.E., Sanz E., McKnight G.S., Stuber G.D., and Palmiter R.D. (2022). Molecular and anatomical characterization of parabrachial neurons and their axonal projections. Elife 11. 10.7554/eLife.81868. PMC9668336 36317965

[116] Campbell J.N., Macosko E.Z., Fenselau H., Pers T.H., Lyubetskaya A., Tenen D., Goldman M., Verstegen A.M., Resch J.M., McCarroll S.A., (2017). A molecular census of arcuate hypothalamus and median eminence cell types. Nat Neurosci 20, 484–496. 10.1038/nn.4495. 28166221 PMC5323293

[117] Schwalbe D.C., Stornetta D.S., Abraham-Fan R.J., Souza G., Jalil M., Crook M.E., Campbell J.N., and Abbott S.B.G. (2024). Molecular organization of autonomic, respiratory, and spinally-projecting neurons in the mouse ventrolateral medulla. J Neurosci. 10.1523/JNEUROSCI.2211-23.2024. PMC11293450 38918066

[118] Gulko A., Essene A., Belmont-Rausch D.M., Veregge M., Pant D., Tenen D., Kapel B.S., Emont M.P., Pers T.H., Rosen E.D., and Tsai L.T. (2024). Protocol for flow cytometry-assisted single-nucleus RNA sequencing of human and mouse adipose tissue with sample multiplexing. Star Protoc 5, 102893. 10.1016/j.xpro.2024.102893. 38416649 PMC10909897

[119] Tao J., Campbell J.N., Tsai L.T., Wu C., Liberles S.D., and Lowell B.B. (2021). Highly selective brain-to-gut communication via genetically defined vagus neurons. Neuron 109, 2106–2115 e2104. 10.1016/j.neuron.2021.05.004. 34077742 PMC8273126

[120] Fleming S.J., Chaffin M.D., Arduini A., Akkad A.D., Banks E., Marioni J.C., Philippakis A.A., Ellinor P.T., and Babadi M. (2023). Unsupervised removal of systematic background noise from droplet-based single-cell experiments using CellBender. Nat Methods 20, 1323–1335. 10.1038/s41592-023-01943-7. 37550580

[121] Kowalczyk M.S., Tirosh I., Heckl D., Rao T.N., Dixit A., Haas B.J., Schneider R.K., Wagers A.J., Ebert B.L., and Regev A. (2015). Single-cell RNA-seq reveals changes in cell cycle and differentiation programs upon aging of hematopoietic stem cells. Genome Res 25, 1860–1872. 10.1101/gr.192237.115. 26430063 PMC4665007

[122] Tyssowski K.M., DeStefino N.R., Cho J.-H., Dunn C.J., Poston R.G., Carty C.E., Jones R.D., Chang S.M., Romeo P., Wurzelmann M.K., (2018). Different Neuronal Activity Patterns Induce Different Gene Expression Programs. Neuron 98, 530 – 546.e511. 10.1016/j.neuron.2018.04.001. 29681534 PMC5934296

[123] Xu S., Hu E., Cai Y., Xie Z., Luo X., Zhan L., Tang W., Wang Q., Liu B., Wang R., (2024). Using clusterProfiler to characterize multiomics data. Nat Protoc 19, 3292–3320. 10.1038/s41596-024-01020-z. 39019974

[124] Boyle E.I., Weng S., Gollub J., Jin H., Botstein D., Cherry J.M., and Sherlock G. (2004). GO::TermFinder--open source software for accessing Gene Ontology information and finding significantly enriched Gene Ontology terms associated with a list of genes. Bioinformatics 20, 3710–3715. 10.1093/bioinformatics/bth456. 15297299 PMC3037731

[125] Todd W.D., Venner A., Anaclet C., Broadhurst R.Y., De Luca R., Bandaru S.S., Issokson L., Hablitz L.M., Cravetchi O., Arrigoni E., (2020). Suprachiasmatic VIP neurons are required for normal circadian rhythmicity and comprised of molecularly distinct subpopulations. Nat Commun 11, 4410. 10.1038/s41467-020-17197-2. 32879310 PMC7468160

[126] Habib N., Zhang F., and Regev A. (2016). Div-Seq: Single-nucleus RNA-Seq reveals dynamics of rare adult newborn neurons. Science 353, 922 – 925. 10.1126/science.aag0863. 27471252 PMC5480621

[127] Moffitt J.R., Bambah-Mukku D., Eichhorn S.W., Vaughn E., Shekhar K., Perez J.D., Rubinstein N.D., Hao J., Regev A., Dulac C., and Zhuang X. (2018). Molecular, spatial, and functional single-cell profiling of the hypothalamic preoptic region. Science 362, eaau5324 – 5314. 10.1126/science.aau5324. 30385464 PMC6482113

[128] Resch J.M., Fenselau H., Madara J.C., Wu C., Campbell J.N., Lyubetskaya A., Dawes B.A., Tsai L.T., Li M.M., Livneh Y., (2017). Aldosterone-Sensing Neurons in the NTS Exhibit State-Dependent Pacemaker Activity and Drive Sodium Appetite via Synergy with Angiotensin II Signaling. Neuron 96, 190–206 e197. 10.1016/j.neuron.2017.09.014. 28957668 PMC5637454

[129] Bankhead P., Loughrey M.B., Fernandez J.A., Dombrowski Y., McArt D.G., Dunne P.D., McQuaid S., Gray R.T., Murray L.J., Coleman H.G., (2017). QuPath: Open source software for digital pathology image analysis. Sci Rep 7, 16878. 10.1038/s41598-017-17204-5. 29203879 PMC5715110

[130] Schindelin J., Arganda-Carreras I., Frise E., Kaynig V., Longair M., Pietzsch T., Preibisch S., Rueden C., Saalfeld S., Schmid B., (2012). Fiji: an open-source platform for biological-image analysis. Nat Methods 9, 676–682. 10.1038/nmeth.2019. 22743772 PMC3855844

[131] Douglass A.M., Resch J.M., Madara J.C., Kucukdereli H., Yizhar O., Grama A., Yamagata M., Yang Z., and Lowell B.B. (2023). Neural basis for fasting activation of the hypothalamic-pituitary-adrenal axis. Nature 620, 154–162. 10.1038/s41586-023-06358-0. 37495689 PMC11168300

